# An Energy Autonomous Microneedle Array‐Based Sensing System for Continuous Biomarker Monitoring

**DOI:** 10.1002/advs.75789

**Published:** 2026-05-22

**Authors:** Arnab Pal, Kai‐Po Fan, Sheng‐Chun Hung, Jaba Roy Chowdhury, Jun‐Hsun Chung, Meenakshi Ray, Hsiang‐Yun Hsu, Fu‐Cheng Kao, Kuei‐Lin Liu, Zong‐Hong Lin

**Affiliations:** ^1^ Department of Biomedical Engineering National Taiwan University Taipei Taiwan; ^2^ Institute of Biomedical Engineering National Tsing Hua University Hsinchu Taiwan; ^3^ Department of Power Mechanical Engineering National Tsing Hua University Hsinchu Taiwan; ^4^ Department of Orthopaedic Surgery Spine Section Chang Gung Memorial Hospital Taoyuan Taiwan; ^5^ College of Medicine Chang Gung University Taoyuan Taiwan

**Keywords:** hybrid power generation, internet of things (IoT), interstitial fluid, microneedle sensors, wearable biosensors

## Abstract

Wearable bioelectronics are revolutionizing personalized healthcare by enabling continuous monitoring of physiochemical indicators. Despite significant advancements, challenges remain in creating platforms that can accurately detect multiple biomarkers while being energy autonomous. This study introduces a wearable, minimally invasive microneedle array platform for real‐time monitoring of clinically relevant biomarkers during exercise. The device features stainless‐steel microneedles (SS‐MNs) with ion‐selective membranes and a glucose‐sensing layer, allowing simultaneous detection of sodium (Na^+^), potassium (K^+^), calcium (Ca^2^^+^), pH levels, and glucose in interstitial fluid (ISF). A triboelectric nanogenerator (TENG) paired with an electromagnetic generator (EMG) harnesses energy from mechanical movements to power the data transmission system, eliminating the need for external power sources. Material characterization confirmed the composition and structure of the ion‐selective membranes, while in vitro electrochemical tests showed excellent sensitivity and selectivity across physiologically relevant concentration ranges. The hybrid power generation system (HPGS), combined with the microneedle array‐based biosensing system (MABS), offers a modern solution for wearable electronics. Additionally, on‐body trials confirmed the platform's ability to continuously track biomarker levels during daily activities. This innovative, minimally invasive system marks a significant advancement in wearable biosensing technology, with potential uses in personalized medicine, chronic disease management, and telemedicine.

## Graphical abstract

This work presents an innovative self‐powered wearable biosensor system for real‐time monitoring of multiple biomarkers in interstitial fluid. The device integrates stainless steel‐based minimally invasive microneedles with ion‐selective membranes, enabling simultaneous Na^+^, K^+^, Ca^2+,^ pH, and glucose detection. A hybrid power generation system (HPGS) integrated into a bicycle wheel harvests energy from motion, powering the sensor and eliminating the need for external power sources. Using the harvested energy, the Bluetooth Low Energy (BLE) module enables convenient data transmission to a mobile interface.

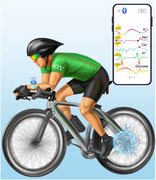

## 1 Introduction

The field of personalized health monitoring is experiencing a revolution driven by rapid advancements in wearable bioelectronic technologies and the Internet of Things (IoT). These innovations enable continuous, real‐time tracking of physical and physiological parameters, opening new horizons in mobile health management [1, 2, 3, 4]. Wireless wearable devices have emerged as powerful tools capable of extracting and transmitting vital health indicators such as pulse, respiration rate, and temperature to user interfaces [5, 6, 7, 8, 9]. However, while these devices excel at measuring physical signals, they face a significant challenge in detecting molecular biomarkers. Traditional biomarker monitoring relies primarily on biofluids such as blood, urine, and saliva, each presenting distinct limitations for continuous wearable sensing applications. Blood sampling, though highly accurate and clinically established, requires invasive venipuncture or finger‐prick procedures that cause discomfort and cannot be practically performed continuously throughout the day [10, 11]. Urine‐based monitoring offers non‐invasive collection but suffers from discontinuous availability, high variability in analyte concentrations due to hydration status, and practical challenges in real‐time sample collection that make it unsuitable for continuous monitoring [12, 13]. Saliva is a more accessible biofluid; however, it exhibits significant variability in composition influenced by eating, drinking, oral hygiene, and circadian rhythms [14, 15], while containing substantially lower concentrations of clinically relevant biomarkers than blood, thereby limiting sensitivity and reliability for continuous monitoring [16, 17].

In this regard, interstitial fluid (ISF) has emerged as a promising alternative that addresses many of these limitations. ISF contains many of the same analytes found in blood, including glucose, lactate, electrolytes, and various metabolites, but can be accessed minimally invasively through microneedle arrays or reverse iontophoresis techniques that penetrate only superficial skin layers [18]. This approach causes minimal discomfort while enabling continuous or frequent measurements. The composition of ISF closely mirrors plasma for many small molecules, providing clinically relevant information without the need for repeated blood draws. Its relatively stable flow, consistent composition, and anatomical accessibility just beneath the skin surface position ISF as an optimal biofluid for wearable sensing platforms designed for long‐term health monitoring applications. Microneedle‐based sensors have demonstrated significant potential for ISF sampling and analysis, offering a minimally invasive approach that penetrates the stratum corneum without causing pain or bleeding [19]. This technology could complement traditional laboratory‐based blood tests [10, 11], enabling the instantaneous monitoring of daily health status and revolutionizing early detection and management of diseases [20, 21, 22]. Recent research has focused extensively on developing novel sensors and improving platform wearability [23, 24]. Despite this progress, several challenges persist in developing wearable biosensors for continuous biomarker monitoring, including achieving high sensitivity and selectivity for multiple analytes simultaneously, ensuring long‐term stability in complex biological environments, and addressing power requirements [25]. Most prototypes have relied on bulky, rigid battery packs to power their electronic circuitry. While flexible batteries and low‐power electronics have been proposed [26, 27]. Batteries still require frequent charging or replacement and raise potential safety concerns. Battery‐free systems powered by near‐field communication (NFC) have been reported [28, 29], but suffer from limited operational range.

This study addresses these challenges by presenting a self‐powered wearable microneedle‐based platform for real‐time monitoring of multiple clinically relevant biomarkers. The device integrates stainless steel microneedles (SS‐MNs) modified with ion‐selective membranes and a glucose sensing layer, enabling simultaneous detection of ions, pH, and glucose in ISF‐analytes chosen for their importance in various physiological processes and relevance to multiple health conditions, including electrolyte imbalances, metabolic disorders, and chronic diseases [30, 31, 32]. A key innovation is the integration of multiple ion‐selective electrodes on a single microneedle platform, representing a significant advancement in wearable biosensing technology. This approach offers several advantages, like simultaneous monitoring of multiple biomarkers, reduced sample volume requirements, and improved spatiotemporal resolution [33]. Beyond improving patient comfort, it enables continuous, real‐time monitoring, particularly valuable for managing chronic conditions and detecting acute health events.

Another crucial part of this work is the energy autonomy that was achieved by utilizing an HPGS. Herein, the HPGS is devised by combining a TENG with an EMG to convert mechanical motion into electrical energy via the coupling of inductive and triboelectric effects [34, 35, 36, 37, 38, 39], offering an energy‐harvesting strategy independent of uncontrollable external sources such as sunlight or wireless power transmitters. This eliminates the need for batteries or external power sources, addressing a major limitation of current wearable devices [33, 34, 35, 36, 37, 38, 39]. In this study, a rotating HPGS system attached to a bicycle wheel generates the required power. The circular movement introduces frictional contact between a static triboelectric disk with a PTFE tribolayer and a rotating Nylon film layer, and produces the required relative movement of the magnets and coils of the EMG. Generated power is stored in a supercapacitor to perform wireless data transfer operations.

By integrating minimally invasive sampling, multi‐analyte detection, and self‐powering features, this wearable microneedle‐based platform marks a significant advancement in biosensing technology. The integrated Bluetooth Low Energy (BLE) module allows easy data transfer to a mobile device for health status monitoring during cycling or other exercises, opening the door to more comprehensive and user‐friendly solutions in personalized medicine, chronic disease management, remote patient monitoring, and telemedicine.

## 2 Results and Discussion

### 2.1 Advanced Wearable Microneedle Array for Multi‐Analyte Self‐Powered Biosensing

**FIGURE 1:**
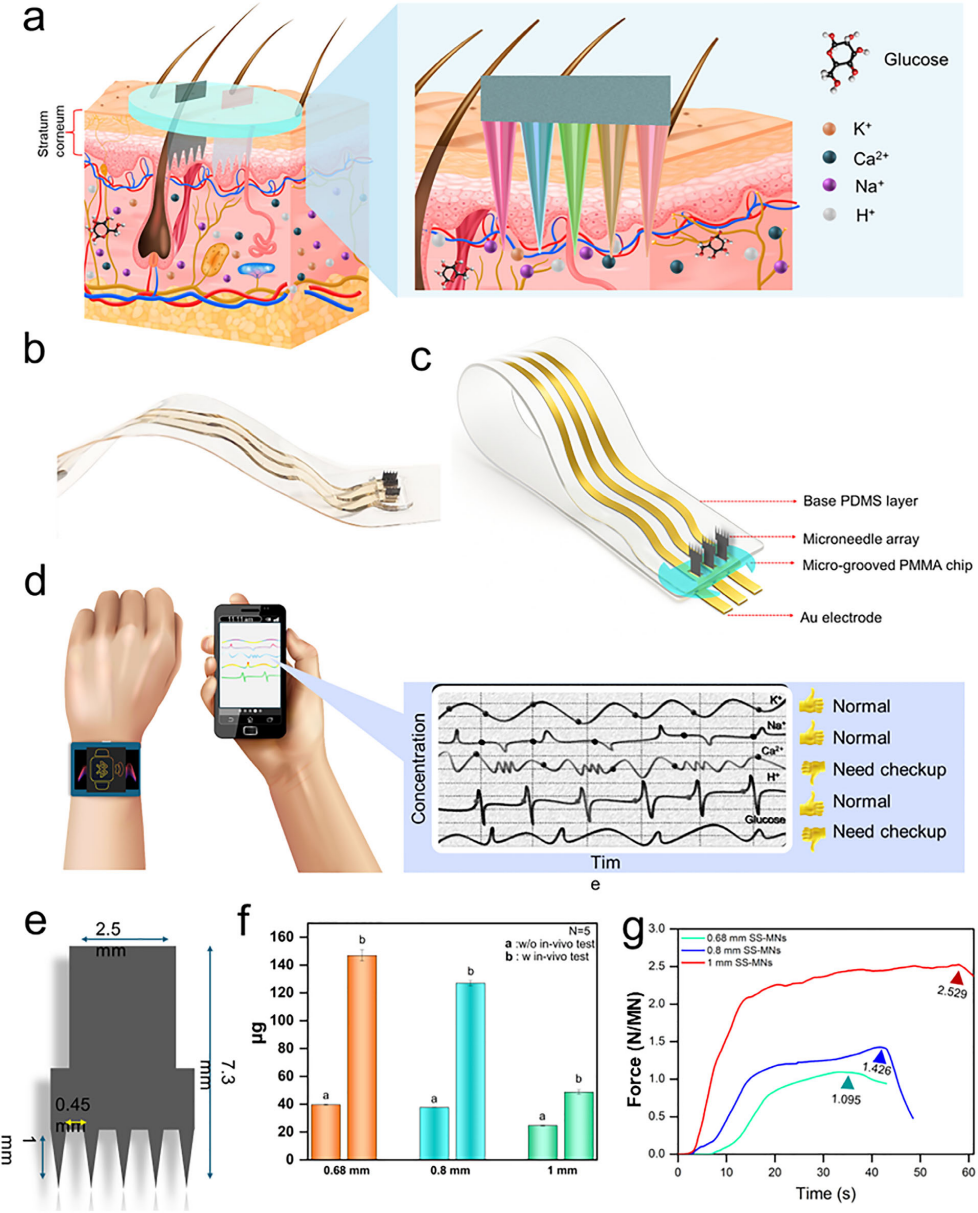
Design and characterization of the wearable microneedle biosensor for continuous metabolic monitoring. (a) Schematic illustration of microneedle‐based interstitial fluid (ISF) sampling from skin (left) and close‐up view showing microneedle penetration through the stratum corneum with real‐time detection of glucose and ions (K^+^, Ca^2^^+^, Na^+^, H^+^) in the dermis. (b) Photograph of the flexible microneedle array device. (c) Exploded view of the multilayer device architecture comprising a base PDMS layer, Au electrodes, micro‐grooved PMMA chip, microneedle array, and protective PDMS top layer. (d) On‐body application showing the wearable sensor mounted on the wrist with wireless data transmission to a smartphone displaying real‐time concentration profiles of multiple biomarkers (K^+^, Na^+^, Ca^2^^+^, H^+^, glucose) with status indicators for physiological assessment. (e) Dimensions of individual microneedles (2.5 mm base width, 0.45 mm tip spacing, 1 mm needle height). (f) Protein contains on microneedle array device (with and without in vivo test), comparing microneedles of different lengths (0.68, 0.8, and 1 mm), showing optimal performance at 1 mm (*n* = 5). (g) Force‐displacement curves demonstrating the mechanical strength of microneedles with varying spacing (0.68, 0.8, and 1 mm), with maximum forces indicated for each configuration.

Schematic cross‐section of human skin illustrates the minimally invasive microneedle array penetrating the stratum corneum and epidermis to access ISF (Figure 1a). The ISF contains various biomarkers of interest, including glucose molecules and ions. This minimally invasive approach enables continuous sampling of physiologically relevant analytes without reaching the pain‐sensing nerve endings located in deeper skin layers. A detailed cross‐sectional view of the specialized microneedles is also presented, each designed to detect a specific analyte: Na^+^, K^+^, Ca^2^^+^, pH, and glucose, from left to right. Specifically, for pH analysis, the deprotonation of H^+^ ions on the surface of the ion‐selective membrane (ISM)‐coated microneedle plays a key role [40]. Each microneedle of the MABS consists of a stainless‐steel core (SS‐MN) coated with a conductive graphene ink layer. An ISM specific to each ion is applied for ion detection, while the glucose‐detecting microneedle includes additional layers of glucose oxidase enzyme and a Nafion protective membrane. This multi‐analyte design allows simultaneous monitoring of several key biomarkers using a single device. The digital image in Figure 1b presents the devised flexible, wearable microneedle array‐based biosensing system. The multilayer architecture of the system is represented in Figure 1c, which includes a base PDMS film, gold electrodes, a micro‐grooved PMMA chip, and a thin protective PDMS layer on top. The developed array serves as the sensing component of a wireless IoT (Internet of Things) system for point‐of‐care health monitoring. A conceptual illustration (Figure 1d) depicts the wearable sensor system attached to a user's wrist, transmitting data wirelessly to a smartphone. The smartphone displays real‐time concentration changes of the monitored biomarkers, with status indicators showing normal levels or alerting users to potential health issues, thereby demonstrating the potential for early disease detection and personalized healthcare monitoring. Dimensional specifications of a single microneedle array are shown in Figure 1e, indicating a length of 1 mm, a base width of 2.5 mm, and a tip diameter of 0.45 mm. These dimensions are optimized to effectively penetrate the stratum corneum while minimizing discomfort and maintaining structural integrity. Additionally, three different microneedle structures were tested to identify the optimal design and dimensions for the most efficient array (Figure S1). The bar graph in Figure 1f compares protein retention on microneedles of varying lengths (0.68, 0.8, and 1 mm) before and after in vivo testing. The data reveal that longer needles (1 mm) exhibit significantly less protein buildup after use, indicating reduced biofouling and potentially improved long‐term sensing stability. In contrast, the 0.68‐ and 0.8‐mm SS‐MNs show a dramatic increase in protein accumulation, likely due to their arrow‐like tips enhancing biofouling effects [41, 42, 43]. The accumulated protein originates from biological fluids and adheres to the SS‐MN surface, reducing the availability of fresh analyte and thereby affecting long‐term sensitivity. Consequently, 1 mm SS‐MNs are considered the most suitable candidate for stable, long‐term monitoring. Furthermore, the force‐time graph (Figure 1g) demonstrates the mechanical strength of microneedles of different lengths. The 1 mm microneedles exhibit superior force tolerance, withstanding up to 2.529 N before failure, compared to 1.426 and 1.095 N for the 0.8 and 0.68 mm needles, respectively. This enhanced mechanical robustness ensures reliable skin penetration and structural integrity during use. Overall, this integrated system represents a significant advancement in wearable biosensing technology, combining minimally invasive sampling, multi‐analyte detection, and robust mechanical performance for self‐powered, real‐time health monitoring.

### 2.2 Characterization of Ion‐Selective Microneedle Sensors

**FIGURE 2:**
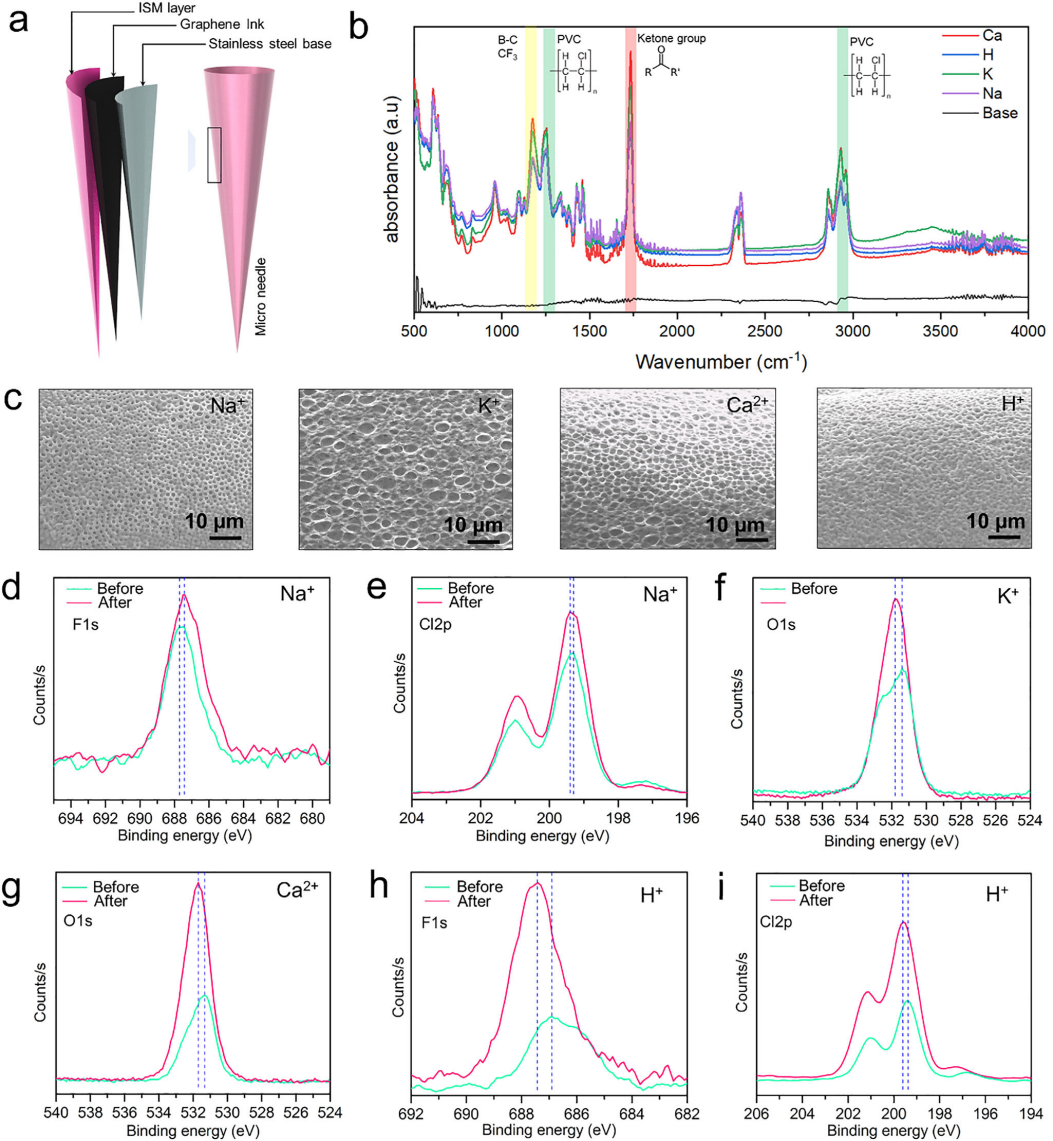
Design, fabrication, and characterization of the microneedle‐based biosensor. (a) Schematic illustration of the microneedle structure with layered components. (b) FTIR spectra of different ion‐selective membranes. (c) SEM images showing surface morphology of ion‐selective membranes for Na^+^, K^+^, Ca^2+^, and H^+^ detection. (d–i) XPS spectra comparing binding energies before and after exposure to target ions for various ion‐selective membranes.

Comprehensive characterization of the ion‐selective microneedle sensors designed for detecting various ions is presented in Figure 2. Each microneedle employs a layered architecture for highly sensitive ion‐sensing via the minimally invasive method. Figure 2a illustrates the schematic diagram of the layered structure, showing the ISM layer as the outer coating, deposited on graphene ink layers, where the graphene ink is applied to the microneedle base. This design aligns with recent advancements in wearable biosensors that utilize advanced conductive nanomaterials like graphene for improved electrical properties and biocompatibility [44]. Fourier Transform Infrared (FTIR) spectroscopy was employed to verify the presence of characteristic functional groups in the ion‐selective membranes (ISMs) designed for specific ion detection (Figure 2b). The spectral analysis revealed several distinctive peaks corresponding to key molecular structures within the ISMs. The PVC matrix exhibited symmetric and asymmetric stretching vibrations of CH_3_ (sp^3^) saturated systems at ∼2858.4 and ∼2940.4 cm^−^^1^, respectively [45, 46], confirming the polymer backbone integrity essential for membrane stability. The presence of PVC was further confirmed by peaks at ∼1420 cm^−^^1^ (C─H aliphatic bond), ∼1250 cm^−^^1^ (C─H bending near Cl), and ∼1031.1 and ∼1097.8 cm^−^^1^ (C─C stretching) [46, 47], validating the membrane's structural framework. Additionally, a band at ∼1020 cm^−^^1^ was attributed to the B─C bond from the ion‐selective component, crucial for ion recognition, while a peak at ∼1733.5 cm^−^^1^ corresponded to C═O stretching (ester) from the plasticizer, which ensures membrane flexibility and ion mobility [48]. These spectral features provide comprehensive evidence for the successful incorporation of all intended functional groups within the ISMs, validating their composition and structure critical for selective ion detection. Besides, Scanning Electron Microscopy (SEM) images reveal the distinct surface morphology of ISM layers for Na^+^, K^+^, Ca^2^^+^, and H^+^ ions (Figure 2c). The varied surface structures observed for different ion‐selective membranes are critical as they directly influence ion diffusion pathways and binding site accessibility, thereby contributing to their specific ion‐sensing capabilities. Recent studies have shown that nanoscale surface features can significantly enhance the sensitivity and selectivity of the sensor [48]. Furthermore, the X‐ray Photoelectron Spectroscopy (XPS) spectra comparing binding energies before and after exposure to target ions are presented in Figure 2d–i. For Na^+^ ISM, F1s and Cl2p spectra are shown (Figure 2d,e), revealing changes in halogen environments upon sodium binding. O1s spectra for K^+^ and Ca^2^^+^ ISMs are displayed in Figure 2f,g, demonstrating oxygen‐mediated coordination with these cations. For H^+^ ISM, F1s and Cl2p spectra are presented in Figure 2h,i, indicating proton‐induced electronic modifications. The observed shifts in binding energies and changes in peak intensities after ion exposure indicate specific interactions between the target ions and their respective selective membranes. These spectral changes provide insights into the ion‐binding mechanisms at the molecular level, which are essential for understanding and optimizing sensor performance through rational membrane design [4, 10]. The combination of these characterization techniques offers a comprehensive view of the MABS, from their macroscopic structure to molecular‐level interactions. The fabrication processes of different ion‐selective membranes are depicted in the supporting information (Figures S2–S5). This multi‐scale characterization approach aligns with recent trends in bioelectronics development [18, 49], where understanding material properties across different length scales is essential for designing high‐performance devices. The tailored surface morphologies observed in the SEM images suggest potential for improved ion selectivity and sensor response times, which are critical factors in real‐time physiological monitoring applications.

### 2.3 Microneedle‐Based Electrochemical Sensing for Real‐Time Monitoring of Physiologically Relevant Biomarkers

**FIGURE 3:**
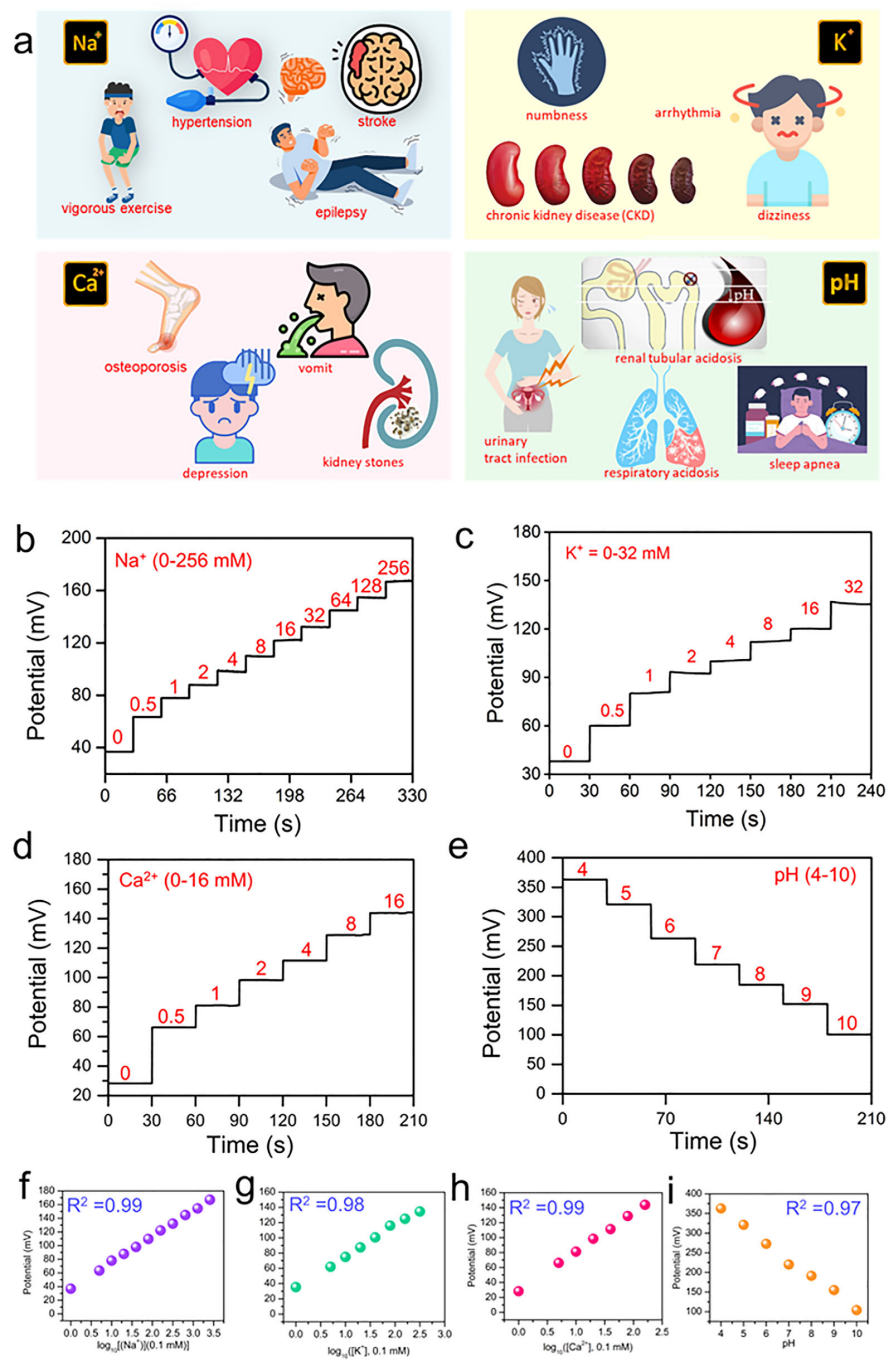
Microneedle‐based electrochemical sensing for monitoring of physiologically relevant biomarkers. (a) Visual overview of health conditions associated with imbalances in Na^+^, K^+^, Ca^2+^, and pH levels. (b–e) The potentiometric responses to increasing concentrations of Na^+^ (0–256 mm), K^+^ (0–32 mm), Ca^2+^ (0–16 mm), and pH changes (4–10). (f–i) Corresponding calibration curves for the sensors, showing excellent linearity with R^2^ values ranging from 0.97 to 0.99.

A visual overview of various health conditions associated with imbalances in key analytes and pH levels in the human body is represented in Figure 3a. First, to evaluate the stability of the fabricated reference electrode, the open‐circuit potential was measured against a commercial Ag/AgCl electrode. The resulting potential stability of the fabricated Ag/AgCl electrode has been reported in Figure S15. These include sodium (Na^+^) linked to hypertension, stroke, and epilepsy [49]; potassium (K^+^) related to arrhythmia, chronic kidney disease, and dizziness [50]; while calcium (Ca^2+^) connected to osteoporosis, depression, and kidney stones [51]; and pH variations associated with urinary tract infections, respiratory acidosis, and sleep apnea [52]. This illustrates the wide‐ranging clinical relevance of these biomarkers in diagnosing and managing multiple health conditions. The real‐time potentiometric responses of the sensors to increasing concentrations of Na^+^ (0–256 mm), K^+^ (0–32 mm), Ca^2+^ (0–16 mm), and pH changes (4–10) are depicted in Figure 3b–e. The step‐like progression in these graphs indicates the sensor's ability to detect discrete changes in analyte levels over time, showcasing its high temporal resolution and sensitivity. These concentration ranges are carefully selected to cover physiologically relevant levels in interstitial fluid, ensuring the sensor's applicability in clinical settings [53, 54, 55, 56]. The corresponding calibration curves are presented in panels of Figure 3f–i, exhibiting excellent linearity with R^2^ values ranging from 0.97 to 0.99 across the tested concentration ranges for each biomarker. This high degree of linearity is crucial for the accurate quantification of analyte concentrations and demonstrates the sensor's reliability across a wide dynamic range. Moreover, to probe the interfacial charge transfer phenomena, the surface potential of the microneedles was measured with the help of Kelvin probe force microscopy (KPFM) for Na^+^, K^+^, Ca^2+^, and H^+^ ISM. In the case of Na^+^, we observe that a 1 mm increase in ion concentration results in about a two‐fold increase in the surface potential, which indicates a decrease in the work function (Figure S6). This suggests that the electrode surface experiences a shift in its local electrostatic environment with the increasing ion concentration. This variation in potential is attributed to interfacial charge redistribution across the interface between the working electrode and the analyte ions in the electrolyte solution, and ion‐membrane interactions, which influence the local electric field. A similar trend is observed in the case of K^+^ ion detection, where the surface potential increases with the increasing concentration of the K^+^ ion (Figure S7). These results very well justify the increasing voltage signal obtained through potentiometry as mentioned earlier. Further, the KPFM analysis for the Ca^2+^ ion, which showed a similar increasing trend with increasing concentration of the analyte (Figure S8). In the case of the H^+^ ion, that is, the varying pH, the surface potential decreased with decreasing H^+^ concentration and increasing pH value (Figure S9). The KPFM results validated the change in the value of the potentiometric signal.

### 2.4 Microneedle‐Based Glucose Sensing for Real‐Time Monitoring

**FIGURE 4:**
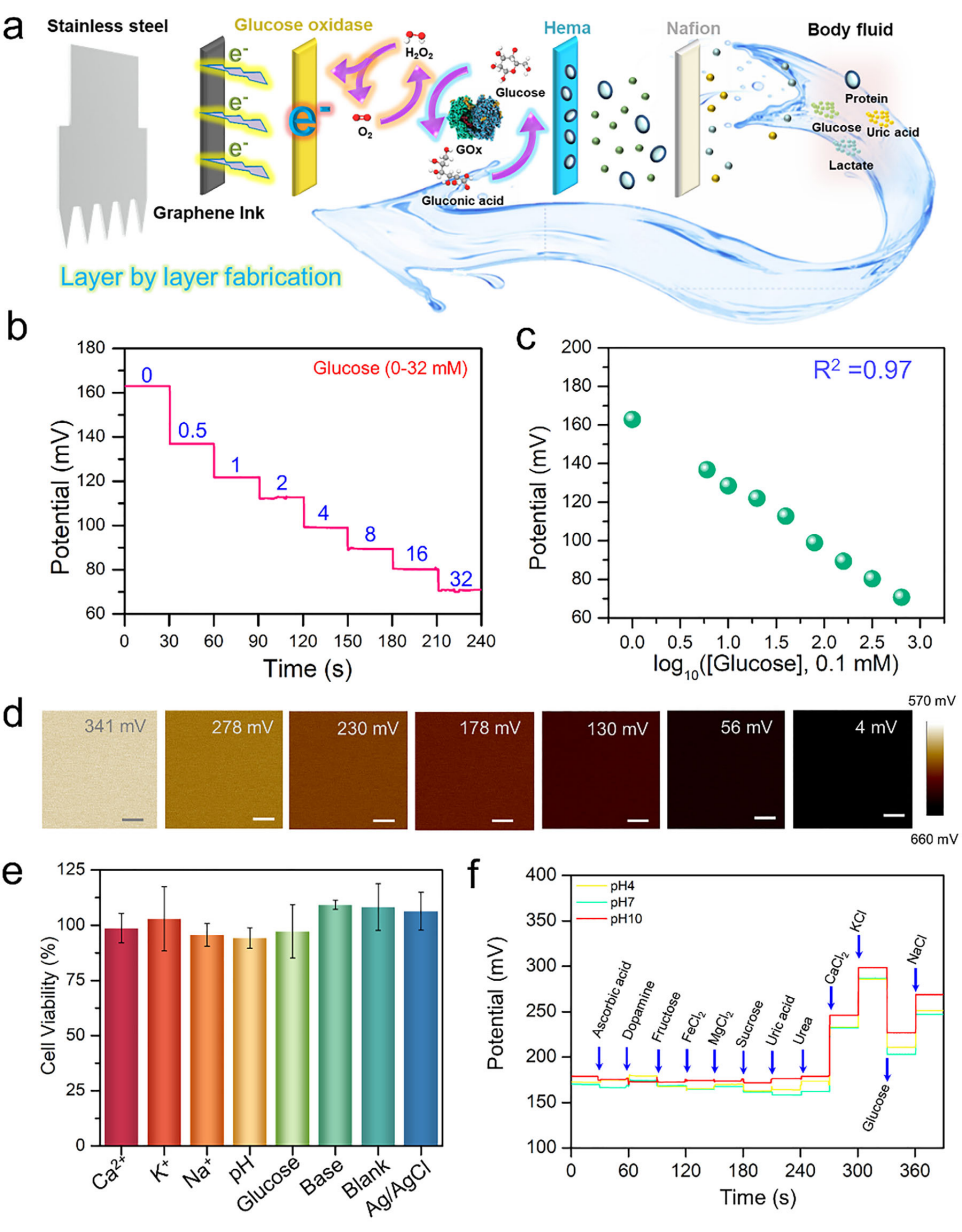
Microneedle‐based glucose sensing for real‐time monitoring. (a) Schematic illustration of the layer‐by‐layer fabrication process for the glucose‐sensing microneedle. (b) Potentiometric response to incrementally increasing glucose concentrations from 0 to 32 mm. (c) Corresponding calibration curve showing linearity (R^2^ = 0.97). (d) Visual representation of the sensor's surface potential changes corresponding to different glucose concentrations. (Scale bar: 100 nm) (e) Cell viability data for different sensor components demonstrating biocompatibility (*n* = 3). (f) Sensor's selectivity demonstrated by response to various potential interfering substances at different pH levels.

The intricate layer‐by‐layer fabrication process of the glucose‐sensing microneedle is illustrated in Figure 4a. The foundation is a stainless‐steel microneedle array, providing structural integrity and the ability to penetrate the skin barrier. This is coated with graphene ink; the subsequent layer consists of glucose oxidase enzymes, which are crucial for the specific detection of glucose molecules [52]. A HEMA (2‐hydroxyethyl methacrylate) hydrogel layer follows, serving as a biocompatible matrix that allows for glucose diffusion while protecting the underlying enzyme layer [53]. However, the outermost Nafion layer acts as a selective membrane, preventing interference from other molecules in body fluids while allowing glucose to pass through [54]. This layered approach to sensor fabrication is designed for both sensitivity and selectivity in glucose detection [55, 56]. The flow chart of the glucose‐sensing microneedle fabrication and the mechanism are represented in Figures S10 and S11. Furthermore, Figure 4b displays the potentiometric response to incrementally increasing glucose concentrations from 0 to 32 mm, covering the physiologically relevant range [52]. The stepwise decrease in potential with increasing glucose concentration indicates a consistent and sensitive response. The corresponding calibration curve shows an extraordinary linearity of 97% (Figure 4c). The linear relationship observed suggests excellent sensor performance across a wide range of glucose levels, which is crucial for accurate monitoring in various physiological states. Furthermore, a visual representation of the surface potential mapping of the sensing surface measured by the KPFM likely corresponds to different glucose concentrations. The color gradient in Figure 4d, from light to dark, represents decreasing surface potential, which is indicative of increasing glucose levels and confirms the change in electron transfer. Moreover, the biocompatibility of the sensing surfaces is addressed in Figure 4e, which presents cell viability data for different sensor components. The high cell viability (close to 100%) across various elements, including those for detecting calcium (Ca^2^^+^), potassium (K^+^), sodium (Na^+^), and glucose, as well as the base materials and reference electrodes (Ag/AgCl), indicates that the sensor is non‐toxic and suitable for biological applications. This is a critical factor for any skin‐interfacing device. Moreover, the skin recovered within an hour after the removal of the microneedles without any wound or irritation, as depicted in Figure S12. Finally, Figure 4f demonstrates the high selectivity of the sensor by showing its response to various potential interfering substances commonly found in body fluids. The minimal change in potential upon introduction of these substances (ascorbic acid, dopamine, fructose, and uric acid) at different pH levels (4, 7, and 10) underscores the ability of the proposed sensor to selectively detect the target ions and glucose even in complex biological environments with varying acidity [52].

### 2.5 Device Structure and Working Principle for Hybrid Energy Harvesting Mechanism Via HPGS

**FIGURE 5:**
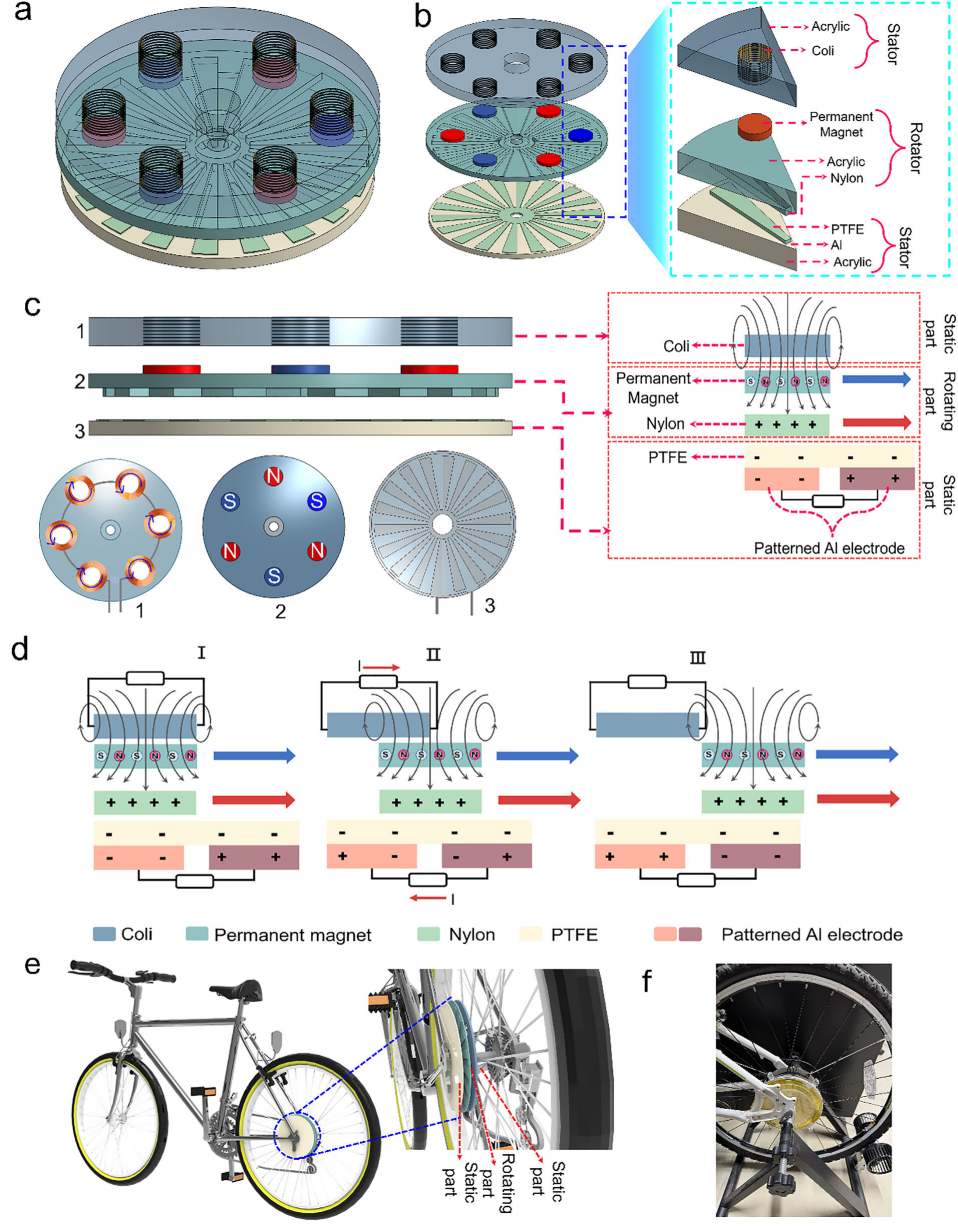
Device structure and working principle of the hybrid power generation system (HPGS). (a) The figure shows an exploded view of the assembled device with its three main disks, (b) the separated top and bottom sections revealing the electromagnetic generator (EMG) with magnets and coils on top and the triboelectric nanogenerator (TENG) with patterned electrodes on the bottom. (c) Detailed cross‐sectional schematics illustrating the layered structure of both the stator and rotating parts. The stator consists of acrylic substrates with embedded coils for the EMG and patterned aluminum electrodes covered with PTFE film for the TENG. The rotating part includes permanent magnets arranged to interact with the coils and a nylon layer to generate triboelectric charges through contact with the PTFE embedded patterned Al electrodes surface. (d) The Schematic diagram of the working mechanism of the HPGS. (e) Representation of the integrated HPGS with the bicycle for hybrid energy harvesting. (f) Digital image of the integrated HPGS with a bicycle.

The hybrid generator integrates two complementary energy conversion mechanisms operating through distinct physical principles to efficiently harvest rotational mechanical energy (Figure 5a,b). The electromagnetic generator (EMG) mechanism is governed by Faraday's law of electromagnetic induction. As depicted in the device structure (Figure 5c), permanent magnets embedded in the rotor rotate relative to stationary copper coils in the stator (Methods and Experimental Section 4.14). During rotation, the magnetic field lines emanating from the magnets sweep through the coil windings, causing continuous variation in magnetic flux linkage. According to Faraday's law, this time‐varying flux induces an electromotive force (EMF) in the coils proportional to the rate of flux change (Figure 5d. **(I)**). When the rotor magnets approach the coils, flux through the coil cross‐section increases, inducing current in one direction. As the magnets move away, the decreasing flux reverses the induced current direction according to Lenz's law, which states that the induced current creates a magnetic field opposing the flux change (Figure 5d **(II)–(III)**). This alternating flux variation produces AC output, with both voltage and current magnitudes scaling directly with rotational velocity, making the EMG particularly effective at higher rotational speeds where rapid flux changes generate substantial induced EMF.

Operating concurrently, the triboelectric nanogenerator (TENG) mechanism exploits the coupling of contact electrification and electrostatic induction through the contact‐separation cycle. The TENG structure consists of a stator with 18 complementary radial aluminum electrodes (0.1 mm thick PET/Al metallized film with Al side as electrode, ∼20° angle each) formed by laser scribing isolation paths on a 2 mm acrylic substrate and covered with a 0.2 mm PTFE dielectric film as the tribonegative layer, and a rotor with 18 fan‐like blades (140 mm outer diameter, 20° angle between blades) laser‐cut from 2 mm acrylic and laminated with 0.02 mm Nylon film as the tribopositive friction layer using 3 m 300LSE double‐sided adhesive. The contact electrification mechanism operates in three states (Methods and Experimental Section 4.13). Herein, in the initial state (Figure 5d **(I)**), when the rotor blade (Nylon surface) contacts the PTFE film, triboelectric charges are generated due to electron affinity differences‐PTFE becomes negatively charged and the Nylon layer becomes positively charged, inducing negative and positive charges on the left and right aluminum electrodes, respectively, through electrostatic induction. During rotation **(II)**, a potential difference develops between electrodes, causing electrons to flow from the left to right electrode through the external circuit to maintain equilibrium. In the final state **(III)**, due to the symmetrical rotary structure, an opposite potential difference is induced as the rotor reaches the opposite position, causing current to flow in the reverse direction, thus generating an AC output. This rotary radial sliding‐mode design with optimized Nylon‐PTFE‐Aluminum electrode combination achieves high voltage output largely independent of rotational speed, while the current remains proportional to the angular velocity, making it ideal for low‐frequency energy harvesting applications. The TENG generates characteristically high voltage (hundreds of volts) with relatively low current (nanoamperes), exhibiting optimal power output at megaohm‐range load resistances, while the EMG produces moderate voltage (single‐digit volts) with higher current (microamperes), matching efficiently to loads in the hundreds of ohms range. This complementarity enables the hybrid system (HPGS) to maintain effective power generation across both low‐frequency conditions where TENG dominates and high‐frequency regimes where EMG becomes more efficient. Together, these design features enable the hybrid system to convert low‐frequency mechanical rotation inputs into a stable electrical output suitable for charging energy storage supercapacitor and powering the BLE module, enabling convenient data transmission for the proposed MABS. Moreover, Figure 5e illustrates the schematic of the HPGS integration with a bicycle for hybrid energy harvesting. A digital image showing the integrated bicycle system for real‐life power generation (Figure 5f). Note S1 presents a calculation‐based analysis of the supercapacitor charging mechanism and energy storage characteristics when powered by the HPGS. Along with a comprehensive comparison table, is provided showing the energy requirements and minimum efficiency for different target voltages used to charge the supercapacitor (Table S1).

### 2.6 The HPGS Harvests Cycling Motion to Energy for Wireless Health Monitoring Sensors and Data Transmission

**FIGURE 6:**
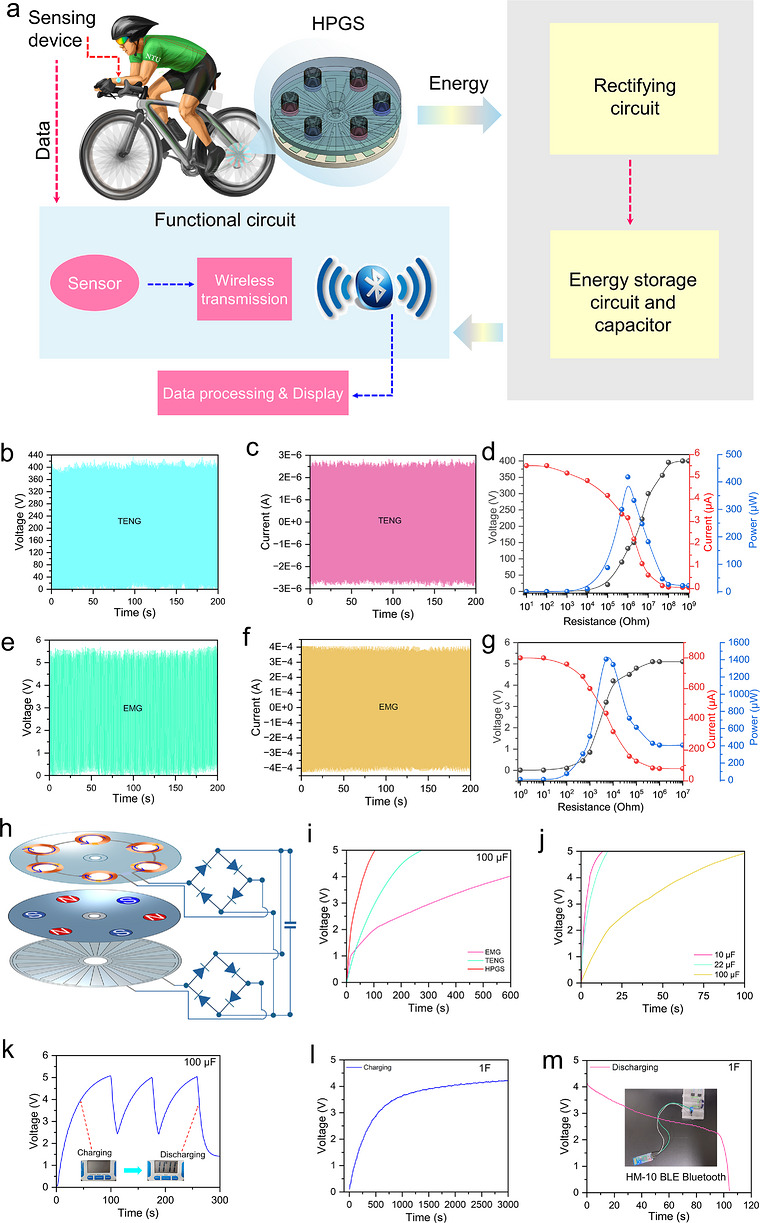
Energy autonomous data transfer system using a hybrid power generation system (HPGS). (a) System architecture showing a cyclist‐worn sensing device powered by harvested mechanical energy through an HPGS generator, including a rectifying circuit and energy‐storage circuits with a 100 µF capacitor that enable wireless data transmission to a data‐processing device with a display. (b–d) Electrical characterization demonstrating TENG generates ∼400 V and ∼5 µA with peak power at 10^6^‐10^7^ Ω resistance. (e–g) EMG produces ∼5.2 V and ∼800 µA with peak power at 10^2^–10^3^ Ω resistance. (h) An equivalent circuit schematic showing TENG and EMG in parallel within the same phase, then rectified separately and connected in parallel to charge the capacitor. (i) Charging curves for the 100 µF capacitor using EMG, TENG, and HPGS. (j) Charging profile of different capacitors using HPGS. (k) Charging and discharging voltage profile of a 100 µF capacitor across multiple consecutive working cycles of a low‐power digital watch. (l) Long‐term charging performance with 1 F capacitor via HPGS. (m) Discharging characteristics with 1 F capacitor powering the BLE module.

The flow chart for the energy‐autonomous monitoring system powering the BLE module for the wearable sensors through a hybrid power generation system harvesting mechanical energy from cycling motion is depicted in Figure 6a. The electrical characterization revealed that TENG generated a voltage of approximately 400 V with a current of ∼5 µA, as shown in Figures 6b and 6c. TENG reached the highest output power optimally at load resistance between 10^6^ and 10^7^ Ω (Figure 6d), displaying distinct voltage and current characteristics compared to EMG generators. Meanwhile, EMG produced a lower voltage of ∼5.2 V but higher current of approximately 800 µA, operating at best power output at 10^2^ to 10^3^ Ω external load (Figure 6e–g). These complementary output characteristics with distinct impedance profiles made TENG and EMG suitable for hybrid integration as the HPGS, through parallel configuration with separate rectification circuits (Figure 6h). The charging performance in Figure 6i demonstrated that HPGS effectively charged the 100 µF capacitor better than individual TENG and EMG configurations alone. The hybrid system showed steeper charging curves, indicating superior power extraction efficiency when combining both TENG and EMG generators in the circuit design. Figure 6j illustrates the charging profiles of different capacitors using HPGS. Practical validation using a low‐power digital watch displayed short‐term charge–discharge cycling of the 100 µF capacitor over multiple operational periods, as shown in Figure 6k. The capacitor voltage oscillated between charging and discharging phases during sensor operation, confirming sufficient energy harvesting from normal cycling activity for continuous operation. Long‐term charging performance with a 1F super capacitor via HPGS is presented in Figure 6l, while Figure 6m shows the discharging characteristics with a 1F capacitor powering the BLE (HM‐10 BLE Bluetooth) module, which is further validated by Video S1. This pattern proves harvested mechanical energy sustains wireless data transmission by the BLE module, which can be integrated with wearable health monitoring applications without requiring external power sources or batteries. The integration of rectifying circuits, energy storage capacitors, and power management successfully enabled self‐powered operation, eliminating battery dependency for wearable sensing devices.

### 2.7 The Wearable System‐Based Multi‐Bio‐Analytes Detection for Real‐Time Monitoring of Physiologically Relevant Biomarkers

**FIGURE 7:**
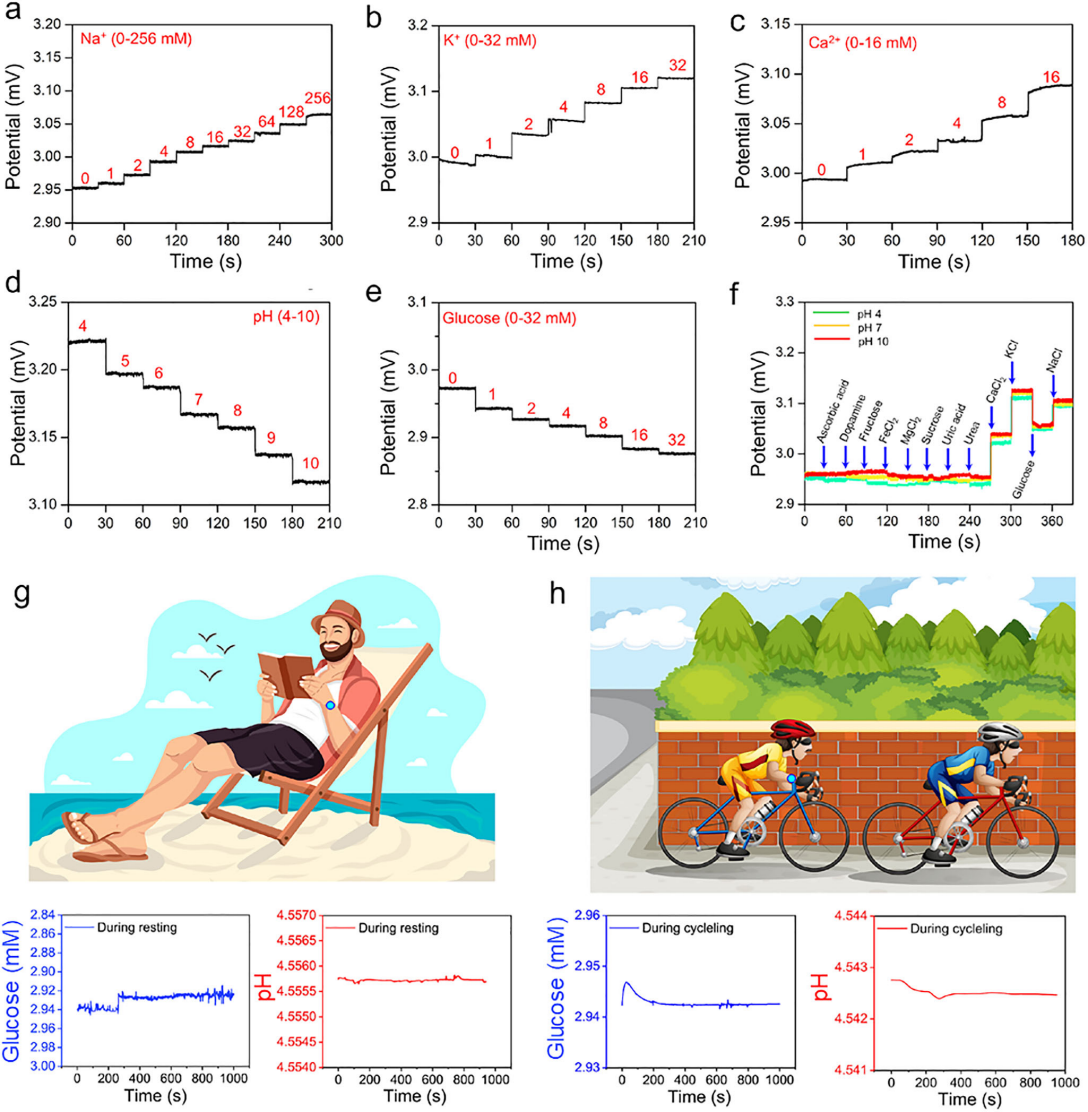
Real‐time biomarker monitoring during different activities using a wearable module. (a–e) Calibration curves showing the potential response to varying concentrations of different biomarkers: (a) Na^+^ (0–256 mm), (b) K^+^ (0–32 mm), (c) Ca^2+^ (0–16 mm), (d) pH (4–10), and (e) glucose (0–32 mm). (f) Selectivity test demonstrating the response to various interfering substances at different pH levels. (g) Real‐time monitoring of glucose concentration and pH levels during resting conditions, with corresponding data plots showing stable measurements over time. (h) Continuous monitoring of glucose and pH levels during cycling activity demonstrating the wearable device's capability to maintain reliable measurements during physical exercise. The results validate the ability to detect biomarkers under different physiological conditions accurately.

The comprehensive calibration of the MABS demonstrated exceptional sensitivity and linearity across all physiologically relevant biomarkers (Figure 7). Na^+^ ion detection exhibited distinct stepwise potential increases from 2.95 to 3.08 mV across the concentration range of 0–256 mm, with clear differentiation between each concentration level (Figure 7a). Besides, K^+^ sensing showed robust potential changes from 2.9 to 3.2 mv for concentrations spanning 0–32 mm, demonstrating excellent resolution within the physiological range (Figure 7b). Moreover, the Ca^2+^ ion monitoring displayed reliable sensitivity from 2.95 to 3.10 mV across 0–16 mm (Figure 7c), successfully covering clinically relevant concentrations. The pH sensor in Figure 7d responded effectively throughout the physiological range from pH 4 to 10, with potentials decreasing systematically from approximately 3.22 to 3.12 mV. In the case of glucose detection, the sensing data showed consistent stepwise potential decreases from 3.0 to 2.8 mV for concentrations ranging from 0 to 32 mm (Figure 7e), encompassing both normal and hyperglycemic levels. Further, the selectivity assessment confirmed the exceptional specificity of the sensor toward their respective target analytes (Figure 7f), with various interfering substances commonly present in biological fluids‐including ascorbic acid, dopamine, fructose, magnesium chloride, sodium chloride, sucrose, lactate, and glucose‐at different pH levels (4, 7, and 10). The potential changes induced by these interfering substances were negligible compared to those produced by the target analytes, confirming excellent selectivity. This robust performance ensures accurate measurements in complex biological environments where multiple compounds coexist with target biomarkers. Furthermore, Real‐time monitoring validation during different physical activity states demonstrated the practical applicability of the wearable sensor system (Figure 7g,h). During resting conditions, both glucose concentration and pH levels remained remarkably stable at approximately 2.94 µm and 4.5550, respectively, with minimal fluctuations throughout extended monitoring periods exceeding 1000 s. These consistent baseline measurements confirm the sensor's stability and reliability during stationary conditions, where metabolic activity remains at basal levels without physical exertion. Continuous monitoring during vigorous cycling activity further validated the system's capability to maintain reliable measurements under dynamic exercise conditions (Figure 7h). Glucose levels exhibited relative stability around 2.95 µm, while pH measurements remained consistent, near 4.542, throughout the entire cycling session spanning over 800 s. The minimal variation observed in these readings during active motion confirms both the wearable device's mechanical robustness and its measurement accuracy under real‐world exercise conditions. The stable signal output despite the physical movements associated with cycling demonstrates effective adhesion of the microneedle array to the skin and resistance to motion artifacts. These comprehensive results validate the energy‐autonomous MABS's ability to detect multiple physiological biomarkers accurately and simultaneously across different activity states, from complete rest to vigorous physical exercise. The performance during both stationary and dynamic conditions demonstrates its suitability for continuous health monitoring applications in real‐world scenarios, enabling seamless integration into daily life activities without compromising measurement accuracy or reliability.

## 3 Conclusion

This study presents an innovative advancement in wearable biosensing through an energy‐autonomous microneedle array‐based sensing system (MABS) enabling continuous, real‐time monitoring of multiple clinically relevant biomarkers. By integrating stainless‐steel microneedles functionalized with ion‐selective membranes and enzymatic glucose‐sensing layers, the platform successfully demonstrates simultaneous detection of Ca^2^^+^, K^+^, Na^+^, pH, and glucose directly from interstitial fluid with exceptional sensitivity and selectivity. A key innovation is the complete energy autonomy achieved through a hybrid power generation system (HPGS) that synergistically combines triboelectric and electromagnetic energy harvesting mechanisms. This system efficiently converts mechanical motion from routine activities into stable electrical energy, eliminating dependence on external power sources or batteries. Comprehensive characterization via FTIR, XPS, SEM, and KPFM confirmed successful fabrication and elucidated charge transfer mechanisms. In vitro electrochemical measurements demonstrated outstanding analytical performance with excellent linearity (R^2^ = 0.97–0.99) across physiologically relevant concentration ranges. The optimized 1 mm microneedles exhibited superior mechanical strength (2.529 N), minimal biofouling, and excellent biocompatibility (>95% cell viability). On‐body validation trials confirmed practical utility during both resting and vigorous exercise conditions, maintaining stable, accurate measurements with minimal motion artifacts. This minimally invasive, self‐powered platform opens transformative possibilities for personalized medicine, chronic disease management, early disease detection, athletic performance optimization, and remote patient monitoring, revolutionizing preventive healthcare and establishing a new paradigm for comprehensive, continuous, accessible health monitoring solutions.

## 4 Methods and Experimental Section

### 4.1 Materials

Stainless steel 316 Medical Grade (Maudlin & Son) was used as the substrate for microneedles. Graphene ink and Ag/AgCl ink were obtained from Hao‐Fu. Glucose Oxidase AC was purchased from AMANO. Other chemicals used include 2‐Hydroxyethyl methacrylate (HEMA), Nafion solution (20%), D‐Glucose (99%, ACROS), sodium hydroxide (97%), magnesium sulfate (>97%, Sigma–Aldrich), sodium hydrogen sulfate (95%), sodium dihydrogen phosphate (99%), sodium chloride (99%–100.5%, SeedChem), potassium chloride (Baker), calcium chloride (96%), sucrose (>99.5%, Sigma–Aldrich), L‐Ascorbic acid (≥98%, Sigma–Aldrich), hydrochloric acid (Honeywell/Fluka), and tetrahydrofuran for HPLC (Sigma–Aldrich). For ion‐selective membranes, the following materials were used: Bis(2‐ethylhexyl) sebacate (≥98%, Tokyo Chemical Industry), sodium tetrakis(3,5‐bis(trifluoromethyl)phenyl) borate (≥98%, Tokyo Chemical Industry), 4‐Tert‐Butylcalix[4]arene‐O, O', O'', O''' (≥96%, Tokyo Chemical Industry), valinomycin crystalline (98%, ACROS), hydrogen ionophore I (ChemCruz), calcium ionophore II (ChemCruz), poly(vinyl chloride) (ALDRICH), and NaTPB (sodium tetraphenylboron). Acrylic sheets (2 mm thickness; outer diameter 140 mm) for backboard, stator, and rotator; double‐sided adhesive (3M 300LSE); nylon film (0.02 mm); PTFE film (0.2 mm); PET/Al metallized film (total thickness 0.1 mm); double‐sided silver conductive tape for lead attachment; NdFeB disc magnets (Ø 30 mm × 3 mm); isopropyl alcohol (IPA); deionized (DI) water. A laser cutter and standard alignment jigs were used for patterning and assembly.

### 4.2 Study Design

This study was conducted under IRB 202500100A3, approved by the Chang Gung Medical Foundation, with written informed consent obtained from all participants prior to enrollment. A total of three healthy adult volunteers from the general population, aged 24–32 years, participated in the on‐body trials. Data collection was conducted at Chang Gung Memorial Hospital. The study followed a controlled design, with the same experimental protocol applied to all participants. Briefly, after skin disinfection with alcohol, a sterilized wearable microneedle sensor was applied to the non‐dominant arm of each participant and secured using a self‐adhesive wrap.

### 4.3 Fabrication of Stainless‐Steel Microneedles (SS‐MNs)

SS‐MNs were fabricated using a laser cutting machine (Laser System DC‐5030‐B) from 0.1 mm‐thick 316 medical standard stainless steel sheets. The microneedles were designed with a length of 1 mm, based on mechanical strength and protein retention studies.

### 4.4 Sterilization of SS‐MNs

SS‐MNs were sterilized by immersion in diluted hydrochloric acid solution (1.2 m) for 5 min, followed by acetone for 15 min, and 75% ethanol for 15 min in an ultrasonic bath cleaner. Finally, the SS‐MNs were exposed to ultraviolet rays (253.7 nm) for 30 min on each side.

### 4.5 Fabrication of Graphene Ink‐Coated Electrodes

SS‐MNs were coated with graphene ink and thermally treated in a circulator oven (DENG YNG DO30). The temperature rose from room temperature to 130°C, held for 30 min, and then slowly decreased to room temperature. The SEM image of the graphene‐coated microneedle is presented in Figure S16.

### 4.6 Fabrication of Ion‐Selective Membranes (ISMs)

#### 4.6.1 Sodium ISM

The sodium ion‐selective membrane (Na+‐ISM) [57, 58, 59, 60] was synthesized following a modified protocol based on the work of Xu et al. [60]. The membrane composition consisted of polyvinyl chloride (PVC, 33 wt.%) as the polymer matrix, sodium ionophore X (1 wt.%) as the ion‐recognition element, sodium tetrakis [3,5‐bis(trifluoromethyl)phenyl] borate (Na‐TFPB, 0.55 wt.%) as the ion‐exchanger, and bis(2‐ethylhexyl) sebacate (DOS, 65.45 wt.%) as the plasticizer [60, 61]. The fabrication process began with dissolving PVC in tetrahydrofuran (THF, 660 µL) under continuous stirring for 16 h to ensure complete solubilization. Subsequently, sodium ionophore X was incorporated into the solution, followed by the addition of Na‐TFPB. The ion‐exchanger plays a crucial role in enhancing the membrane's selectivity toward sodium ions by facilitating the discrimination between cations and anions. Finally, DOS was introduced as a plasticizer to improve the mechanical properties of the membrane and promote the integration of the ionophore within the PVC matrix. The plasticizer acts as a molecular bridge, maintaining the overall electrochemical equilibrium of the membrane. Upon completion of the mixing process, the resulting ISM solution was stored at 4°C to preserve its integrity and stability until further use (Figure S2).

#### 4.6.2 Potassium ISM

The preparation of K^+^ ion‐selective membranes follows the method described in [57, 58, 59, 60]. To create these membranes, PVC (33 wt.%), which serves as the matrix, is first dissolved in cyclohexanone (350 µL) and stirred for 16 h. Subsequently, potassium ionophore I (valinomycin) (2 wt.%) is added while continuing to stir. Sodium tetraphenyl‐boron (NaTPB) (0.5 wt.%) is then incorporated as an ion additive, which helps distinguish between cations and anions, providing basic selectivity for the target ion. Following this, bis(2‐ethylehexyl) sebacate (DOS) (64.7 wt.%) is added as a plasticizer [60, 61]. The plasticizer's primary function is to soften the PVC membrane and facilitate its integration with the ionophore, acting as a bridge between membrane components and maintaining overall electrochemical equilibrium. Once prepared, the entire ISM solution is stored at 4°C for future use (Figure S3).

#### 4.6.3 Calcium ISM

The preparation of Ca^2+^ ion‐selective membranes is conducted following the protocol delineated in [57, 58, 59, 60]. The process begins with the dissolution of PVC (33 wt.%), which constitutes the membrane's structural foundation, in tetrahydrofuran (THF) (660 µL). This mixture undergoes continuous agitation for 16 h. Subsequently, bis(2‐ethylehexyl) sebacate (DOS) (65.5 wt.%) is introduced as a plasticizing agent [60, 61], which enhances the flexibility of the PVC membrane and promotes its amalgamation with the ionophore. The next step involves the addition of calcium ionophore II (ETH 129) (1 wt.%) while maintaining constant agitation. Following this, sodium tetrakis [3,5‐bis(trifluoromethyl)phenyl] borate (Na‐TFPB) (0.5 wt.%) is incorporated as an ionic enhancer. This compound facilitates the differentiation between cations and anions, conferring specific selectivity toward the target ion and functioning as a molecular liaison within the membrane, thereby preserving the overall electrochemical balance. Upon completion of the synthesis, the resultant ISM solution is preserved at a temperature of 4°C for subsequent utilization (Figure S4).

#### 4.6.4 Hydrogen ISM

The fabrication of H^+^ ion‐selective membranes commences with the dissolution of PVC (33 wt.%), serving as the membrane's structural framework, in tetrahydrofuran (THF) (660 µL) [57, 58, 59, 60]. This mixture undergoes continuous agitation for 16 h. Subsequently, hydrogen ionophore I (tridodecylamine) (1 wt.%) is incorporated while maintaining constant stirring. The next step involves the addition of sodium tetrakis [3,5‐bis(trifluoromethyl)phenyl] borate (Na‐TFPB) (0.55 wt.%) as an ionic enhancer. This compound plays a crucial role in discriminating between cations and anions, thereby imparting fundamental selectivity toward the target ion. Following this, bis(2‐ethylehexyl) sebacate (DOS) (65.45 wt.%) is introduced as a plasticizing agent [60, 61]. The primary function of this plasticizer is to enhance the flexibility of the PVC membrane and facilitate its integration with the ionophore, effectively acting as a molecular bridge within the membrane structure. This process ensures the maintenance of overall electrochemical equilibrium. Upon completion of the synthesis, the resulting ISM solution is stored at a temperature of 4°C for future applications (Figure S5).

### 4.7 Fabrication of Glucose Sensing Membrane

The glucose‐sensing membrane was fabricated using a layer‐by‐layer approach. First, graphene ink was coated on the SS‐MNs. Then, a glucose enzyme mixture was uniformly coated and cured at 37°C for three cycles. Finally, a Nafion protective layer was applied [62] (Figure S10).

### 4.8 Immobilization Condition of Glucose Oxidase

The bicinchoninic acid (BCA) assay, a widely accepted method for protein quantification, was employed to validate the enzyme content and assess inter‐group variations following SS‐MNs fabrication [57, 58, 59, 60]. This colorimetric technique relies on the reduction of Cu^2+^ to Cu^+^ by proteins in an alkaline environment, followed by the chelation of Cu^+^ with two BCA molecules, resulting in a color transition from apple green to purple. The intensity of this chromogenic reaction is directly proportional to the protein concentration. Absorbance measurements were conducted at 562 nm using a plate reader, and protein content was determined via a standard bovine serum albumin (BSA) calibration curve. As illustrated in Figure S11, 10 sets of enzyme‐coated SS‐MNs samples exhibited visually consistent purple hues, suggesting comparable protein concentrations. Quantitative analysis revealed that the enzyme content across all 10 sets of enzyme‐coated microneedles fell within a narrow range of 20–30 micrograms. This consistent distribution not only confirms the successful immobilization of the enzyme layer on the microneedles but also demonstrates the uniformity of the coating process across multiple samples.

### 4.9 Reference Electrode Fabrication

The reference electrode was fabricated by coating SS‐MNs with Ag/AgCl ink using a 30G needle tip, followed by thermal treatment in a circular oven.

### 4.10 Electrochemical Measurements

Electrochemical measurements were performed using a CHI 6205E Electrochemical Analyzer in open circuit potential (OCPT) mode. A two‐electrode system was used, with the working electrode being the SS‐MNs coated with ISM or glucose‐sensing membrane, and the reference electrode being the Ag/AgCl‐coated SS‐MNs.

### 4.11 Surface Potential Mapping and Work Function Calculation

The work function of the samples was determined by measuring the contact potential difference (CPD) between a conductive diamond AFM tip (AD‐2.8‐AS single crystal) and the sample surface. The CPD relates to the work functions of the tip (Φ_tip_) and sample (Φ_sample_) according to:

(1) $$\mathrm{CPD}=\left(\Phi_{\mathrm{tip}}-\Phi_{\mathrm{sample}}\right)/e$$

where “e” is the elementary charge.

The tip was calibrated using highly oriented pyrolytic graphite (HOPG) as a reference standard (Φ_HOPG_ = 4.6 eV) [63, 64], yielding a tip work function of 5.00 eV. The sample work function was then calculated from the measured CPD values using:

(2) Φsample=Φtip−e×CPDsample

This approach enables precise determination of surface work function, providing insights into the electronic properties and surface potential of the material [65]. To ensure statistical reliability, measurements were performed on at least three independent sample sets for each amino acid, with a minimum of three measurement positions per sample [66].

### 4.12 Characterization

Field Emission Scanning Electron Microscopy (FESEM, JEOL JSM‐7900F) was used to characterize the surface morphology of the SS‐MNs. Fourier Transform Infrared (FTIR) spectroscopy (L1600400 Spectrum, PerkinElmer) and X‐ray Photoelectron Spectroscopy (XPS, ULVAC‐PHI PHI 5000 Versaprobe III) were used to analyze the chemical composition of the ISMs.

### 4.13 Cytotoxicity Analysis

The cytotoxicity assessment of SS‐MNs is crucial for in vivo applications due to their complex chemical composition. To evaluate SS‐MN cytotoxicity, a comprehensive cellular assay was conducted using NIH 3T3 fibroblasts, a well‐established cell line derived from NIH Swiss mice. Cryopreserved NIH 3T3 cells were resuscitated and cultured in Dulbecco's Modified Eagle Medium (DMEM) supplemented with 10% Fetal Bovine Serum and 1% Penicillin‐Streptomycin. After 24 h, the medium was replenished, and cell morphology was evaluated using a Nikon ECLIPSE Ts2 optical microscope to confirm uniform distribution. Concurrently, SS‐MNs underwent a 48‐h immersion in DMEM within 96‐well plates to extract potential leachates. The extracted leachate was then introduced to cultured NIH 3T3 cells and incubated for 24 h in a CO_2_ incubator at 37°C and 5% CO_2_. Cell viability was quantified using the Cell Counting Kit‐8 (CCK‐8), a colorimetric assay where tetrazolium salt is reduced by dehydrogenases in viable cells to form a soluble, orange formazan dye [67]. After a 1‐h incubation with CCK‐8, the absorbance of each well was measured using a SpectraMax iD3 microplate reader. The quantity of formazan produced is directly proportional to the number of living cells, providing a reliable measure of cell viability. This methodology ensures a robust evaluation of the potential cytotoxic effects of SS‐MNs, providing critical data to inform further in vivo studies and the development of safe, biocompatible microneedle‐based sensing platforms.

### 4.14 Fabrication of the TENG

Backboard, stator, and rotator substrates were patterned from 2 mm acrylic by laser cutting according to the CAD layout (outer diameter 140 mm). The rotator featured a 21 mm center bore to fit the common shaft interface. All acrylic parts were cleaned with IPA and DI water and dried with nitrogen prior to lamination. For the Rotator part having the fan‐like blade structure with 18 blades at 20° angle with each other, the acrylic rotator disc was uniformly laminated with 3 m 300LSE double‐sided adhesive, followed by bonding a 0.02 mm nylon film as the tribopositive layer (Figure S13). The triboactive region and registration features were defined by laser cutting. The finished rotator was aligned and bonded to the backboard using the center bore and fiducial features to maintain coaxiality with the stator during assembly. For the Stator part, A PET/Al metallized film (Al side serving as the electrode; 0.1 mm total thickness) was laminated onto the acrylic stator substrate. Complementary “positive/negative” electrodes were formed by laser scribing isolation paths to obtain two electrically separated electrode sets. Lead‐outs for each electrode were connected using double‐sided silver conductive tape to minimize contact resistance without thermal loading. A 0.2 mm PTFE film was then laminated on top as the tribonegative layer, and access windows for terminals/alignment were opened by laser cutting. Care was taken to avoid wrinkles and trapped air to maximize the effective contact area.

### 4.15 Fabrication of the EMG

For the Stator part, an acrylic disc (outer diameter 140 mm; thickness 2 mm) served as the substrate. Six circles were laser‐cut on one face at angular positions registered to the rotor magnets. Pre‐wound coils—each comprising 50 turns of enamelled copper wire (0.2 mm conductor diameter)—were embedded into the grooves and fixed with insulating adhesive. For the rotator, six NdFeB disc magnets (each measuring 30 mm in diameter and 3 mm in thickness) with the same pole facing outward were uniformly arranged and fixed on one face of an acrylic rotor disc (outer diameter 140 mm; thickness 2 mm). Magnet angular positions were registered to the stator coil positions to enhance magnetic coupling and induced EMF (Figure S13).

### 4.16 Electrical Measurement

The output voltage and the current signal of the TENG+EMG (HPGS) via a voltage preamplifier (Keithley 6514 System Electrometer) were acquired. The software platform was constructed based on LabVIEW, which was capable of realizing real‐time data acquisition, control, and analysis. Further, the wireless signal transfer HM‐10 HM10 Bluetooth 4.0 BLE Module is used, which is run by using a super capacitor of 1F (KR‐5R5V105‐R), which is charged by the HPGS (Figure S14).

### 4.17 Data Analysis

Interference studies were conducted using various physiologically relevant molecules, and the relative response was calculated.

### 4.18 Statistical Analysis

Statistical analyses were performed using Origin software. The sample size (n) used for statistical analysis was 3–5 according to experimental requirements. Data were presented as mean ± SD.

## Author Contributions

Z.‐H. L. conceived the research idea, acquired funding, and supervised the entire project. A. P. and K.‐P. F. contributed equally to this work. A. P., K.‐P. F., and Z.‐H. L. conceived and designed the experiments. A. P., K.‐P. F., S.‐C. H., J. R. C., J.‐H. C., M. R., H.‐Y. H., F.‐C. K., and K.‐L. L. performed the experiments and collected the data. A. P., K.‐P. F. analyzed the data and interpreted the results under the guidance of Z.‐H. L. A. P. wrote the manuscript. Z.‐H.L. revised the manuscript. All authors discussed the results and commented on the manuscript.

## Conflicts of Interest

The authors declare no conflicts of interest.

## Supporting information




**Supporting File 1**: advs75789‐sup‐0001‐SuppMat.docx.

**Supporting File 2**: advs75789‐sup‐0002‐VideoS1.mp4.

## Data Availability

The data that support the findings of this study are available from the corresponding author upon reasonable request.
