# Supplementary material for: An Energy Autonomous Microneedle Array‐Based Sensing System for Continuous Biomarker Monitoring
Source: Adv Sci (Weinh). 2026 May 22:e75789. Online ahead of print. doi: 10.1002/advs.75789 (PMC13335777; doi:10.1002/advs.75789)
Supplement: Supplementary file 1 — Supporting File 1: advs75789‐sup‐0001‐SuppMat.docx. [file ADVS-9999-e75789-s001.docx]

Supporting Information

**An Energy Autonomous Microneedle Array-based Sensing System for Continuous Biomarker Monitoring**

Arnab Pal ^a,1^, Kai-Po Fan ^a,b,1^, Sheng-Chun Hung ^a^, Jaba Roy Chowdhury ^a^, Jun-Hsun Chung ^b^, Meenakshi Ray ^c^, Hsiang-Yun Hsu ^a^, Fu-Cheng Kao ^d,e^, Kuei-Lin Liu ^a,b^, Zong-Hong Lin ^a,c,^*

^a^ Department of Biomedical Engineering, National Taiwan University, Taipei 10167, Taiwan

^b^ Institute of Biomedical Engineering, National Tsing Hua University, Hsinchu 30013, Taiwan

^c^ Department of Power Mechanical Engineering, National Tsing Hua University, Hsinchu 30013, Taiwan

^d^ Department of Orthopaedic Surgery, Spine Section, Chang Gung Memorial Hospital, Taoyuan 33305, Taiwan

^e^ College of Medicine, Chang Gung University, Taoyuan 33302, Taiwan

*Corresponding author at: zhlin@ntu.edu.tw

Arnab Pal ^1^, Kai-Po Fan ^1^: These authors contributed equally to this work.

**CONTENTS**

**Supporting Note**

**Supporting Note 1.** Theoretical analysis of supercapacitor charging by the HPGS

**Supporting Tables**

**Table S1.** Comparison of energy requirements and minimum efficiency for different target voltages

**Supporting Figures**

**Figure S1.** Dimensional illustration of SS-MNs.

**Figure S2.** Fabrication procedure of sodium ionophore X-based sodium ISM

**Figure S3.** Fabrication procedure of Valinomycin-based potassium ISM

**Figure S4.** Fabrication procedure of ETH129-based calcium ISM

**Figure S5.** Fabrication procedure of Tridodecylamine-based hydrogen ISM

**Figure S6.** Surface potential mapping and work-function calculation of the SS-MNs sensor at different Na⁺ ion concentrations.

**Figure S7.** Surface potential mapping and work-function calculation of the SS-MNs sensor at different K⁺ ion concentrations.

**Figure S8.** Surface potential mapping and work-function calculation of the SS-MNs sensor at different Ca²⁺ ion concentrations.

**Figure S9.** Surface potential mapping and work-function calculation of the SS-MNs sensor at different pH values.

**Figure S10.** Fabrication procedure of glucose sensing membrane

**Figure S11.** Immobilization condition of the Gox.

**Figure S12.** Skin reaction following sensor patch removal.

**Figure S13.** Fabrication and assembly of the hybrid power generation system (HPGS).

**Figure S14.** Experimental circuit configuration demonstrating supercapacitor charging and wireless BLE module functionality

**Figure S15.** Potential variation of the as-fabricated reference electrode in simulated ISF, the open-circuit potential versus a commercial Ag/AgCl reference electrode (3M NaCl) was measured

**Figure S16.** SEM images of the microneedles showing the full coverage of the needle tip

**Supporting Note 1.** **Theoretical analysis of supercapacitor charging by the HPGS.**

In our hybrid energy harvesting system, the supercapacitor charging performance was analyzed for a target voltage of 4.0 V. The energy required to charge the 1 F supercapacitor to this voltage was calculated using the capacitive energy equation:

E_req_ = ½CV²……………………………………..(1)

E_req_ = 0.5 × 1 × 4² = 8.00 J

The total power output from the hybrid system, combining both TENG and EMG sources, was determined by:

P_tot_ = P_TENG_ + P_EMG_ = V_TENG_·I_TENG_+ V_EMG_·I_EMG_……………………………………..(2)

P_to t_= (400 × 5×10⁻⁶) + (5.2 × 800×10⁻⁶)

P_tot_ = 2.0 + 4.16 = 6.16 mW

Over the experimental duration of 3000 seconds, the total energy theoretically available from both sources was:

E_avail_ = P_tot_ × t……………………………………..(3)

E_avail_ = 0.00616 × 3000 = 18.48 J

The minimum end-to-end system efficiency required to achieve the 4.0 V target within this timeframe was calculated as:

η_min_ = E_req_ / E_avail_……………………………………..(4)

η_min_ = 8.00 / 18.48 = 0.433 ≈ 43.3%

This represents the fraction of generated energy that must be successfully captured and stored after accounting for all system losses including rectification losses, power conversion inefficiencies, impedance mismatch losses, and supercapacitor leakage. The relationship between charging time and system efficiency follows:

t = E_req_ / (P_tot_ × η) ……………………………………..(5)

For comparison, a comparative analysis of different target voltages is presented in Table 1, which demonstrates the significant advantage of selecting a lower target voltage.

**Table S1:** Comparison of energy requirements and minimum efficiency for different target voltages

| **Target Voltage (V)** | **Energy Required (J)** | **Minimum Efficiency (%)** |  |
| --- | --- | --- | --- |
| 5.0 | 12.50 | 67.66 |  |
| 4.0 | 8.00 | 43.29 |  |

The comparative analysis demonstrates that achieving a 4.0 V charge requires only 43.3% efficiency, which is readily attainable with practical energy harvesting systems. In contrast, the 5.0 V target would demand 67.7% efficiency, requiring significantly more sophisticated power electronics. This 24.4 percentage point reduction in efficiency requirement makes the system highly feasible for real-world implementation. The achieved 4.0 V output is sufficient to reliably operate the Bluetooth Low Energy (BLE) module, thereby validating the hybrid energy harvesting approach for self-powered wearable sensor applications during cycling activities.


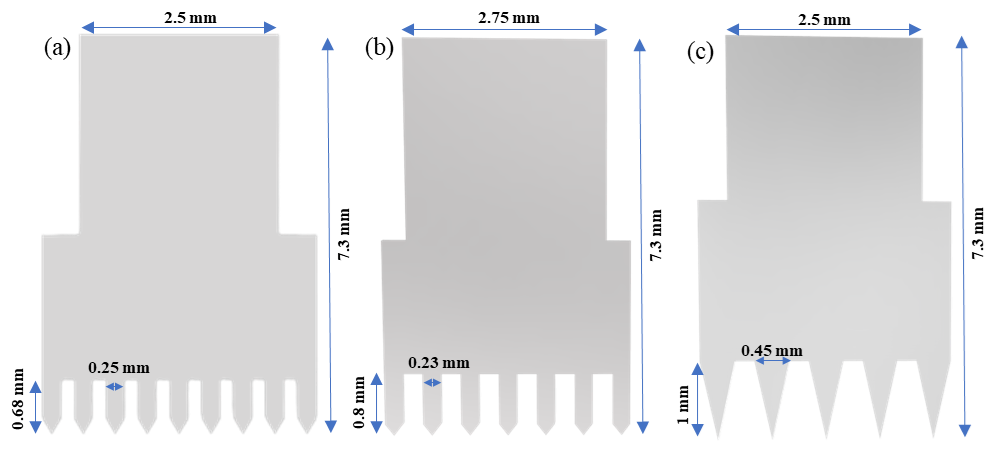


**Figure S1**. Dimensional illustration of SS-MNs (a) design of 0.68 mm (b) 0.8 mm (c) 1mm needle-length SS-MNs.


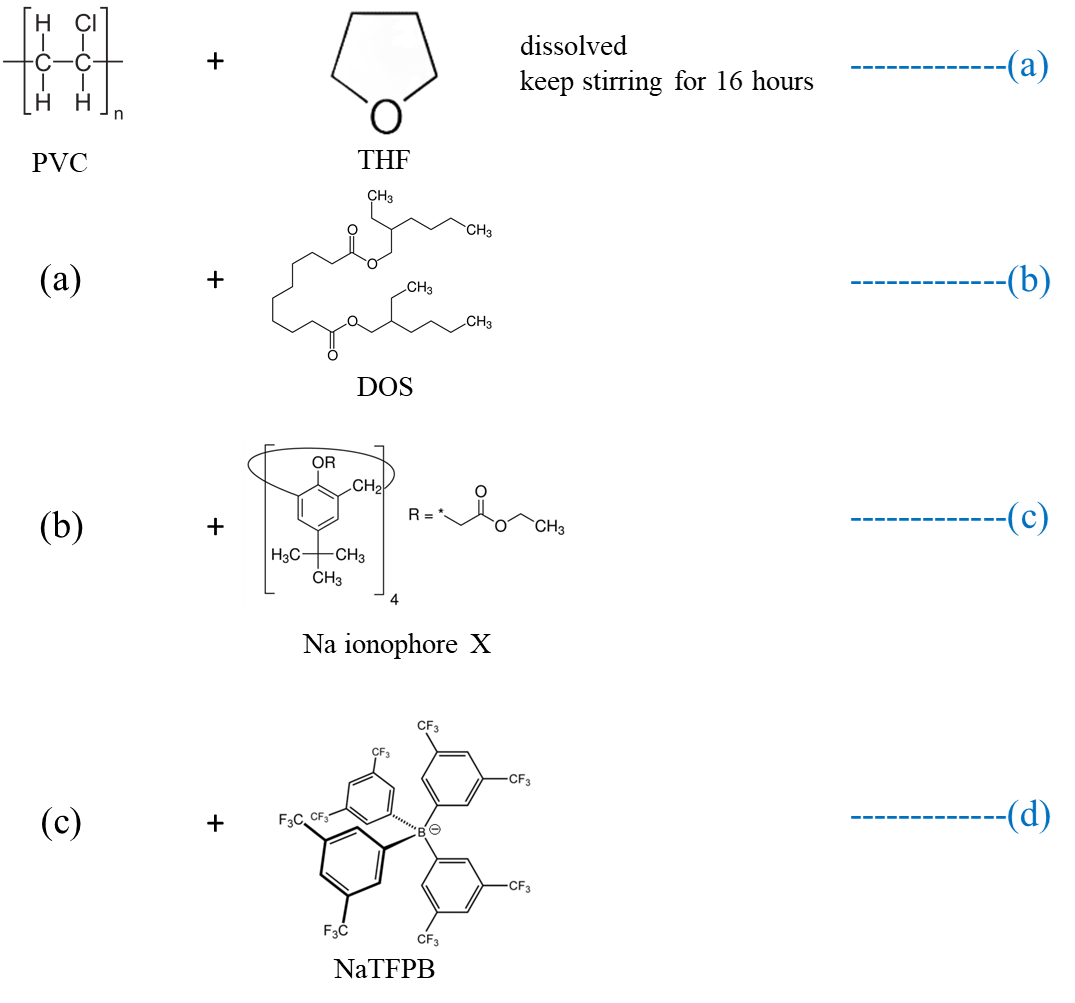


**Figure S2.** Fabrication procedure of sodium ionophore X-based sodium ISM.


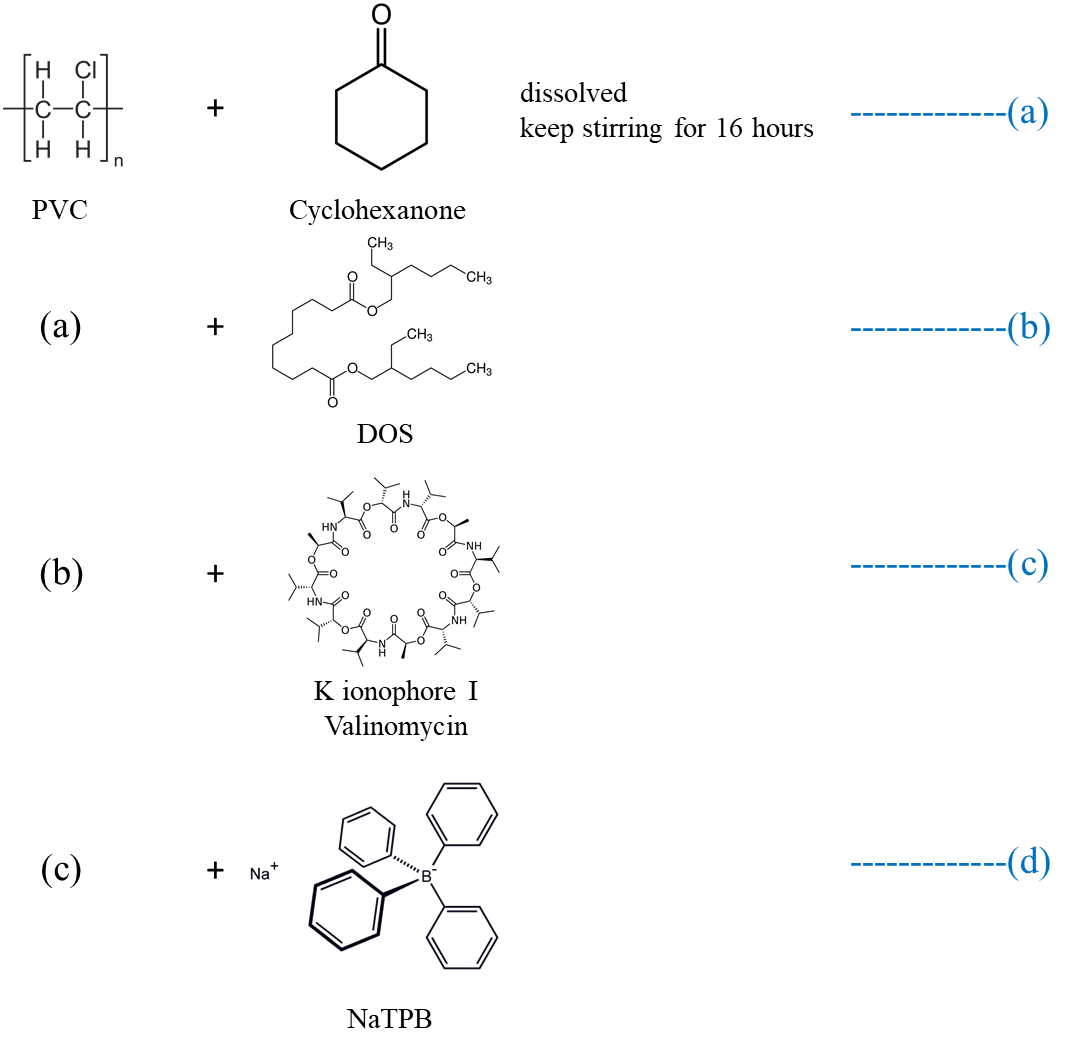


**Figure S3**. Fabrication procedure of Valinomycin-based potassium ISM.


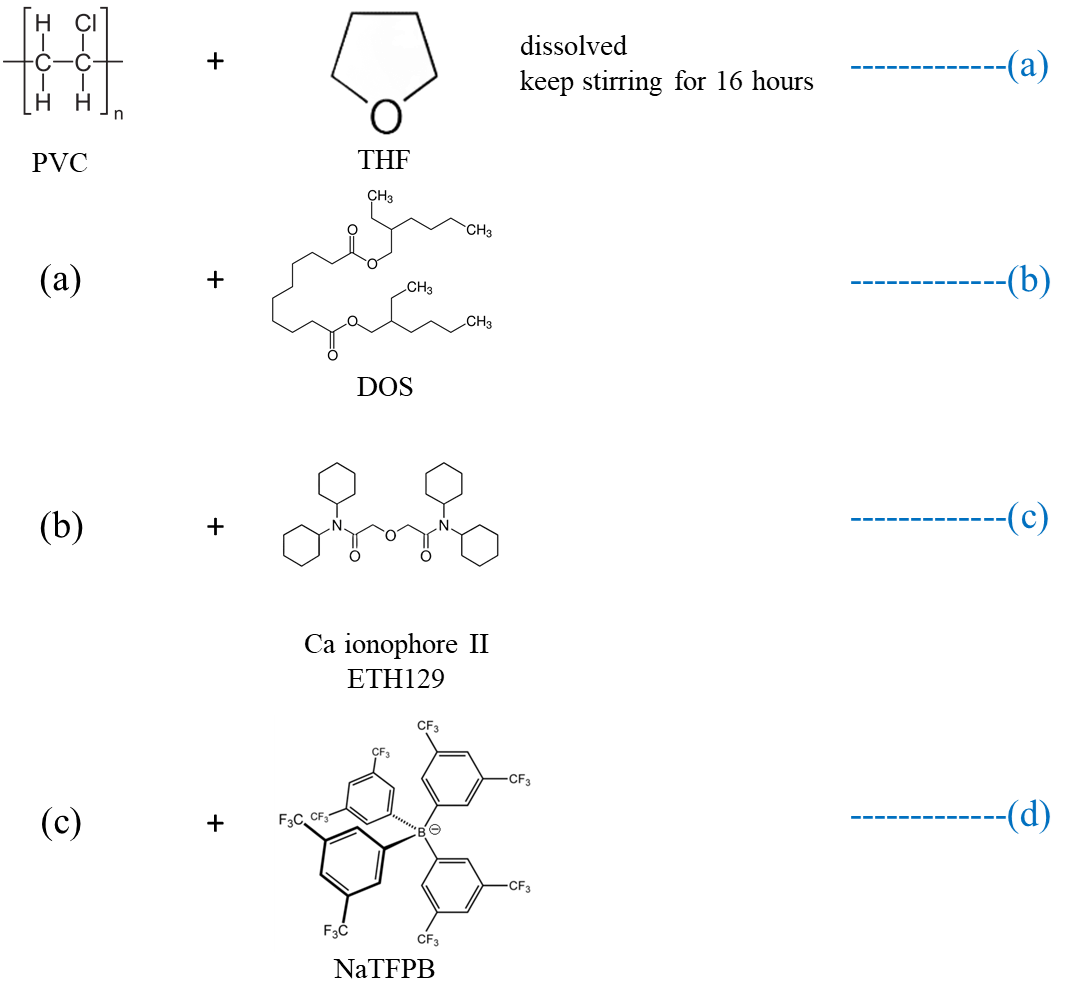


**Figure S4**. Fabrication procedure of ETH129-based calcium ISM.


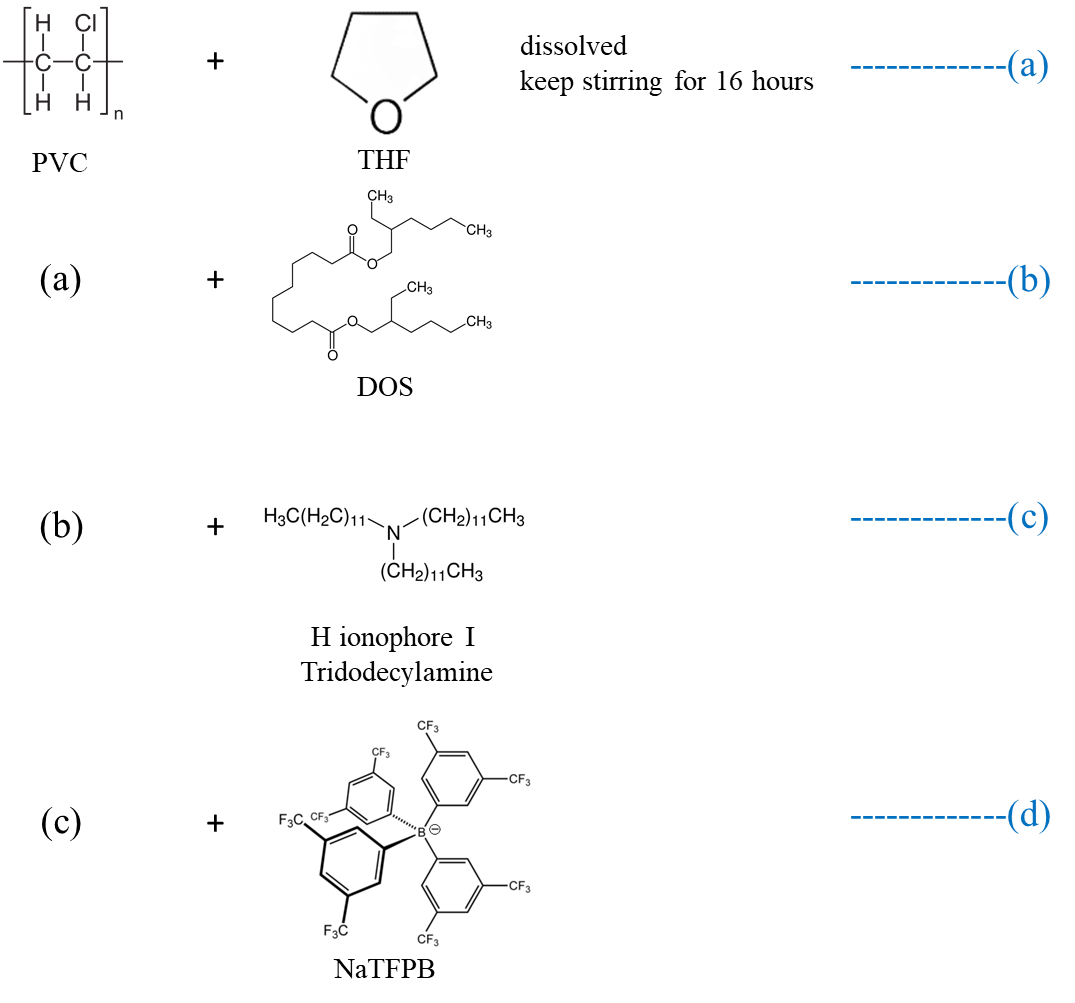


**Figure S5**. Fabrication procedure of Tridodecylamine-based hydrogen ISM.


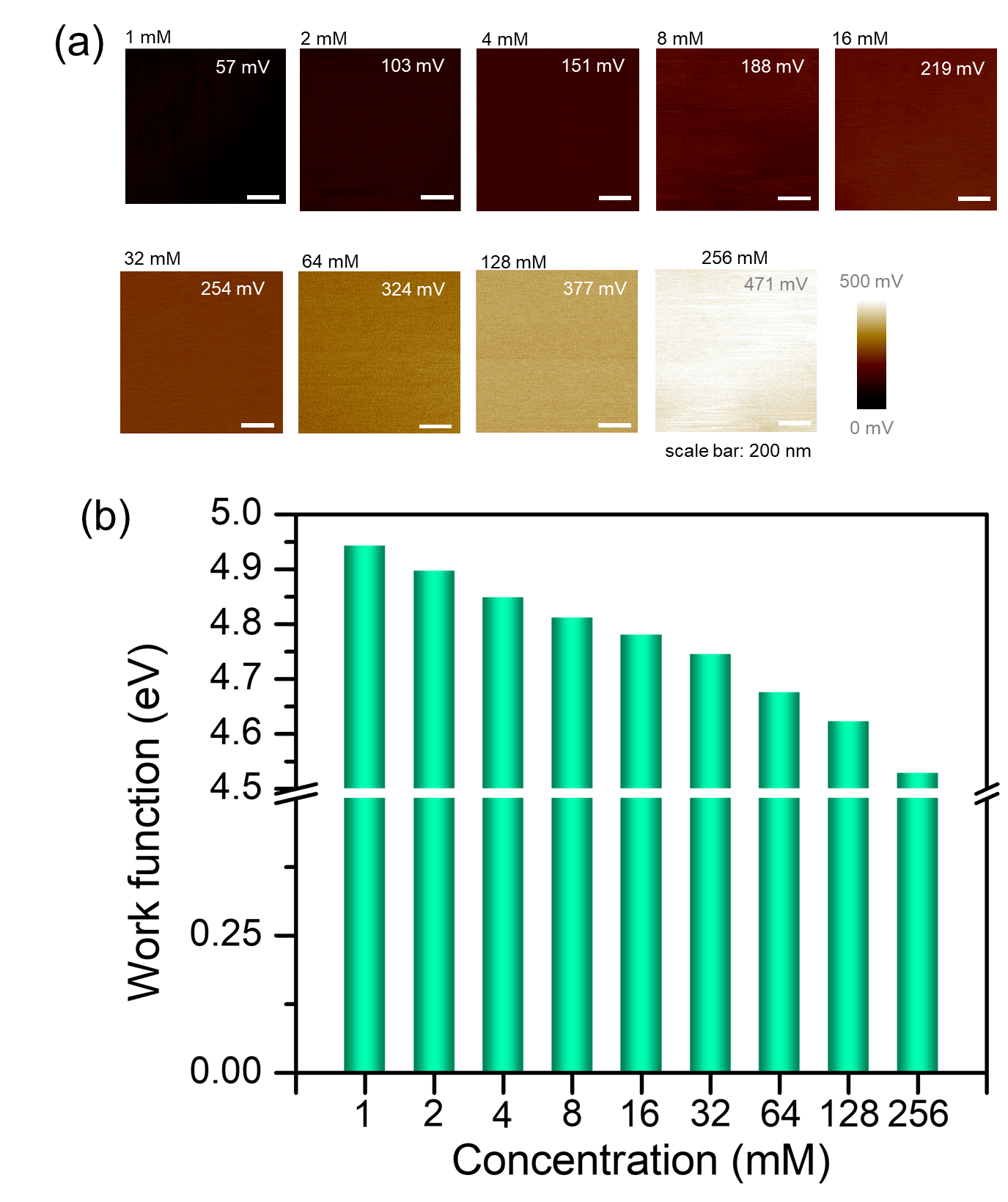


**Figure S6.** (a) Surface potential mapping of the SS-MNs sensor at different Na^+^ ion concentrations. (b) The corresponding work function values at different concentrations of the Na^+^ ion.


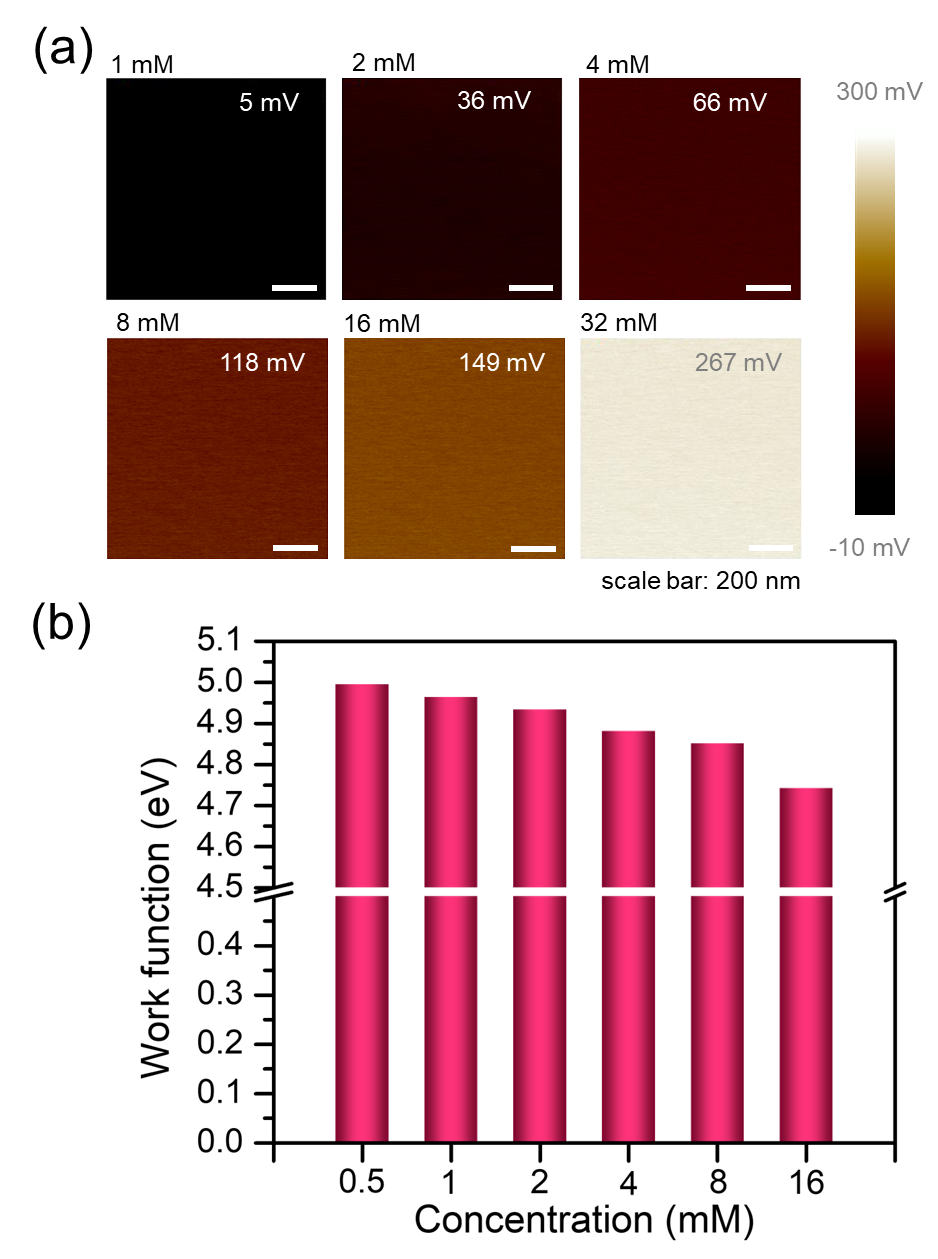


**Figure S7**. (a) Surface potential mapping of the SS-MNs sensor at different K^+^ ion concentrations. (b) The corresponding work function values at different concentrations of the K^+^ ion.


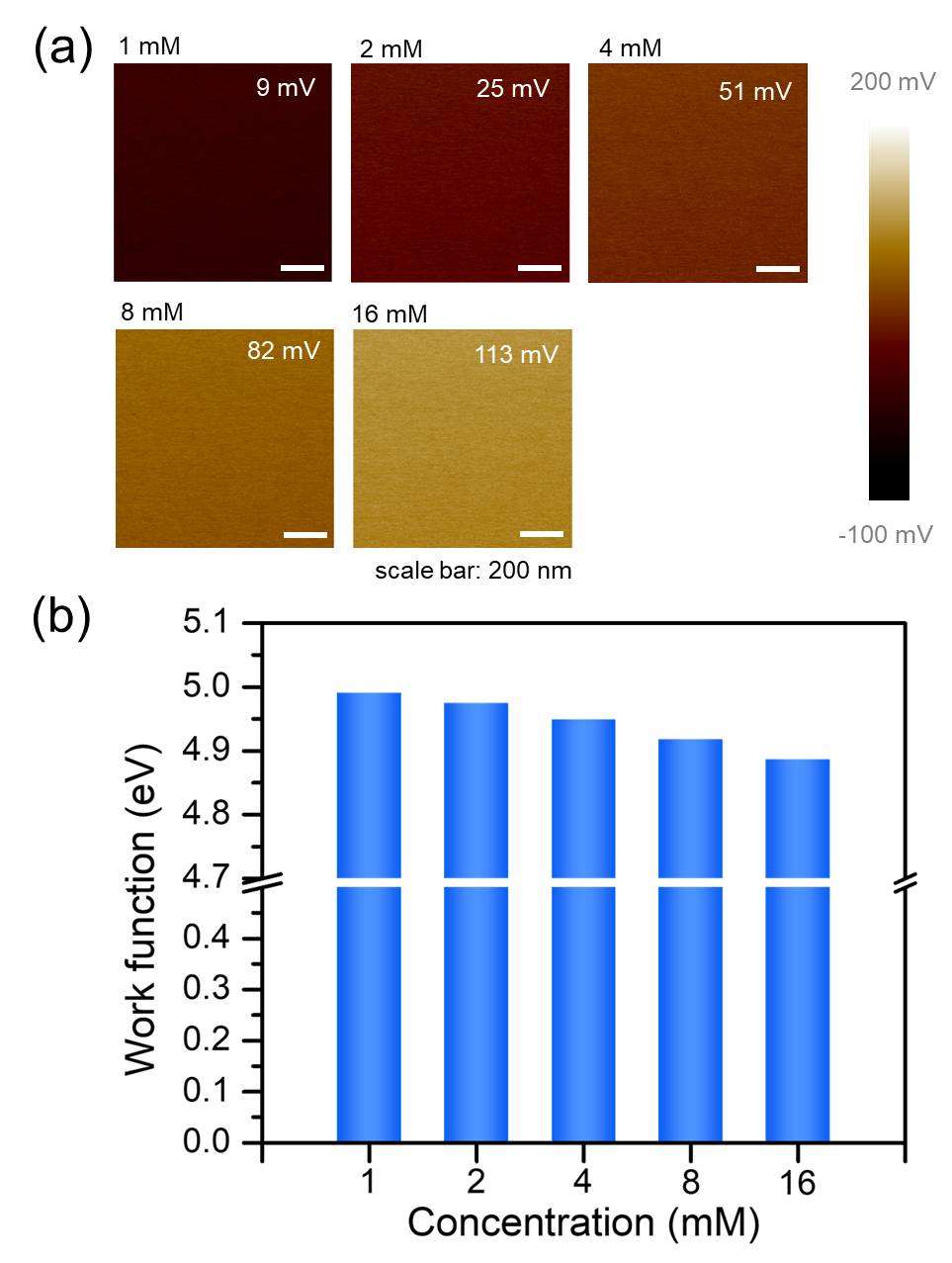


**Figure S8**. (a) Surface potential mapping of the SS-MNs sensor at different Ca^2+^ ion concentrations. (b) The corresponding work function values at different concentrations of the Ca^2+^ ion.


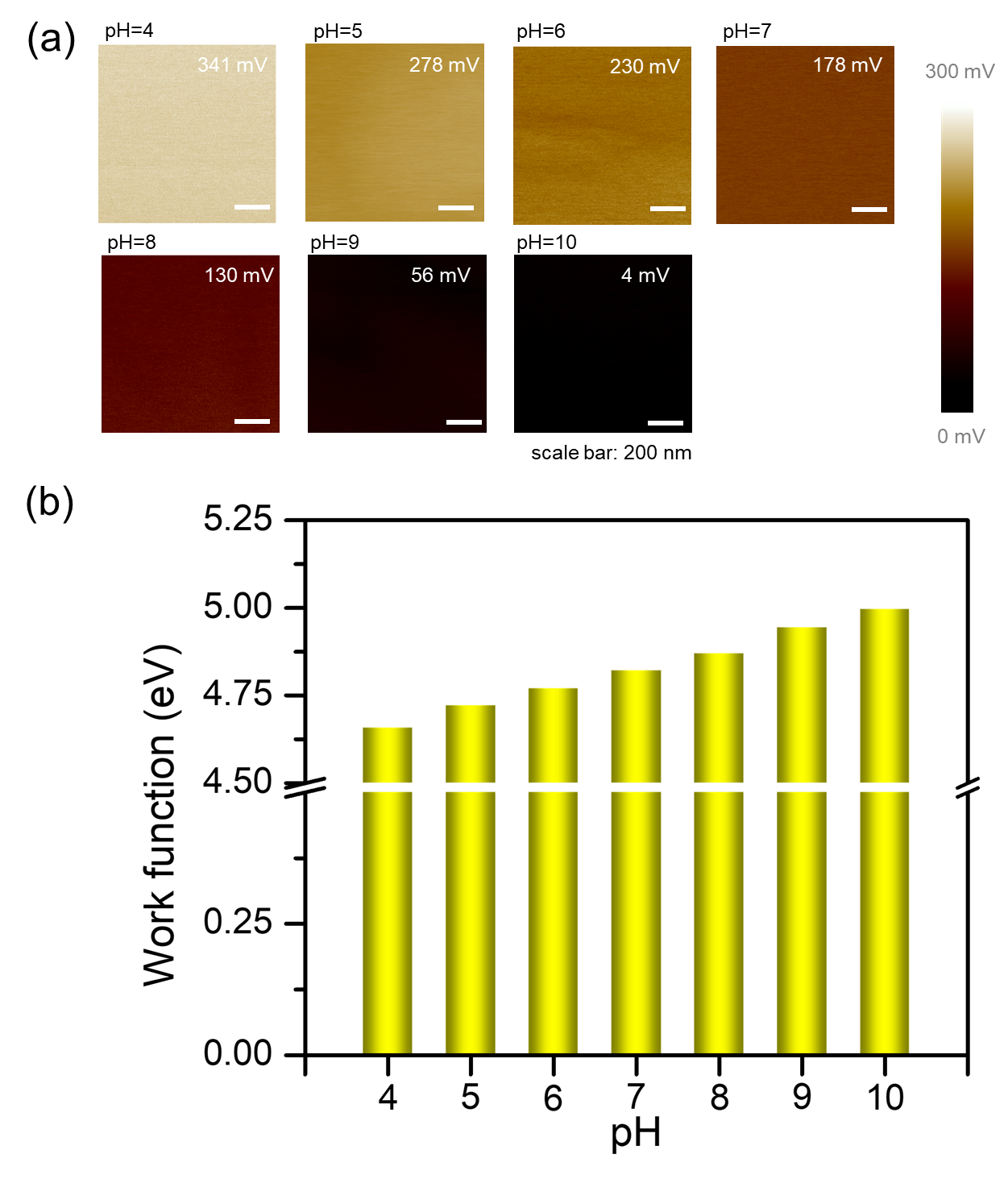


**Figure S9**. (a) Surface potential mapping of the SS-MNs sensor at different pH values. (b) The corresponding work function values at different pH values.


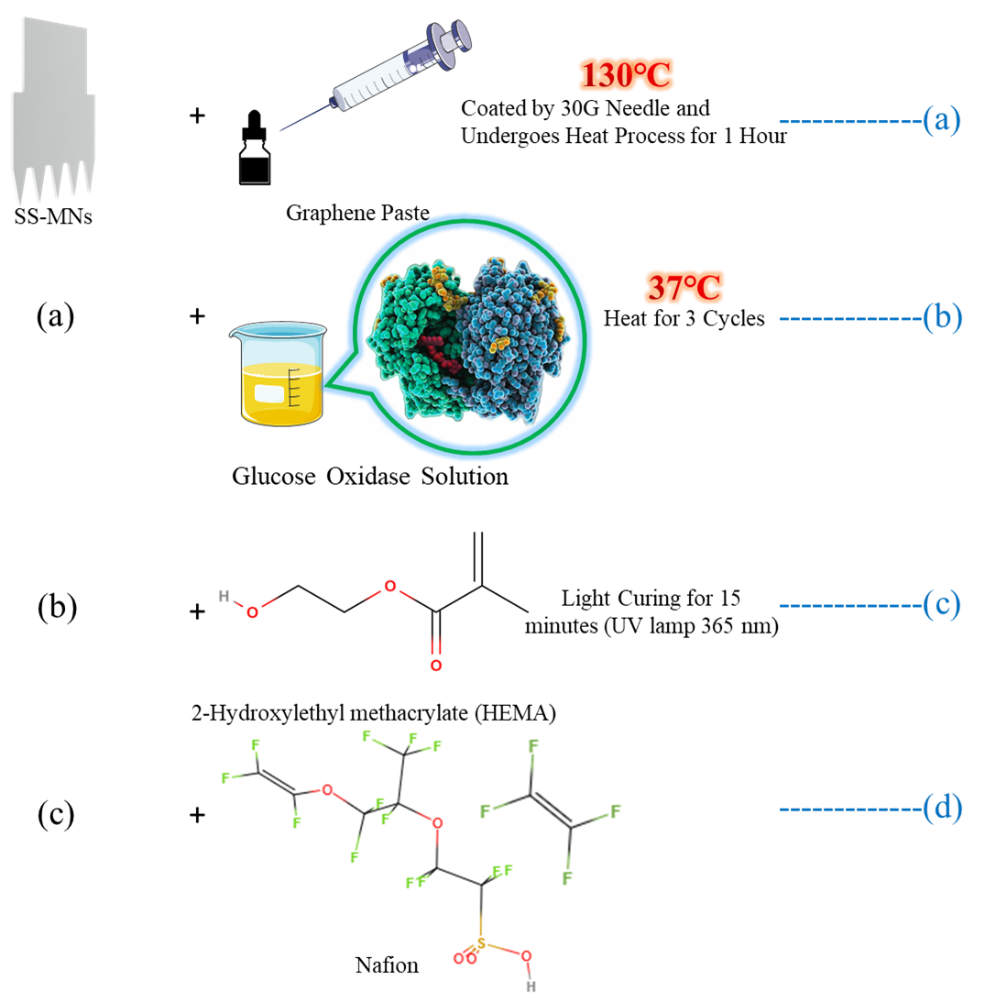


**Figure S10**. Fabrication procedure of glucose sensing membrane.


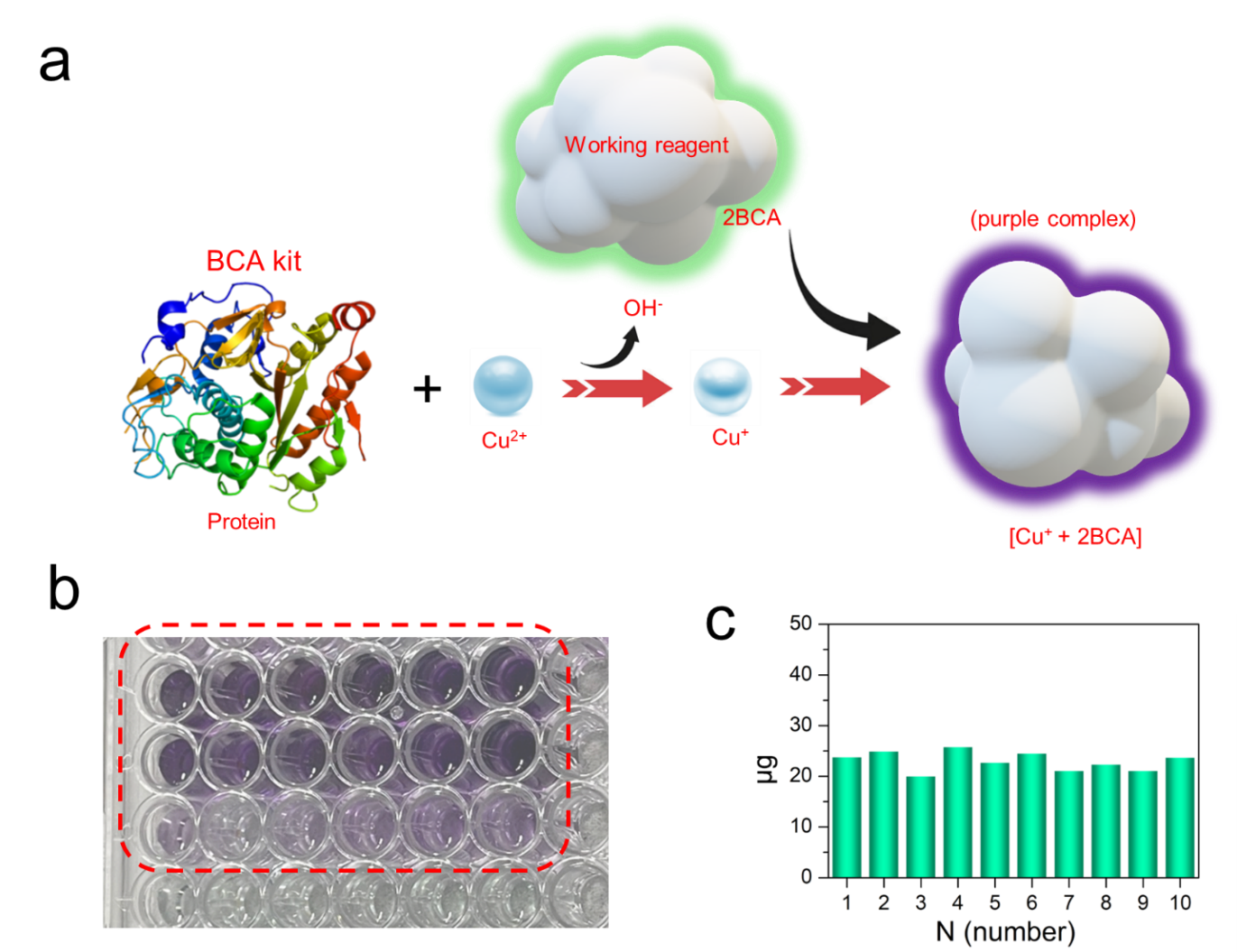


**Figure S11**. Immobilization condition of the GOx (a) working principle of BCA kit. (b) Outlook of SS-MNs in 96 wells. (c) Calculated result of the protein contents on SS-MNs.


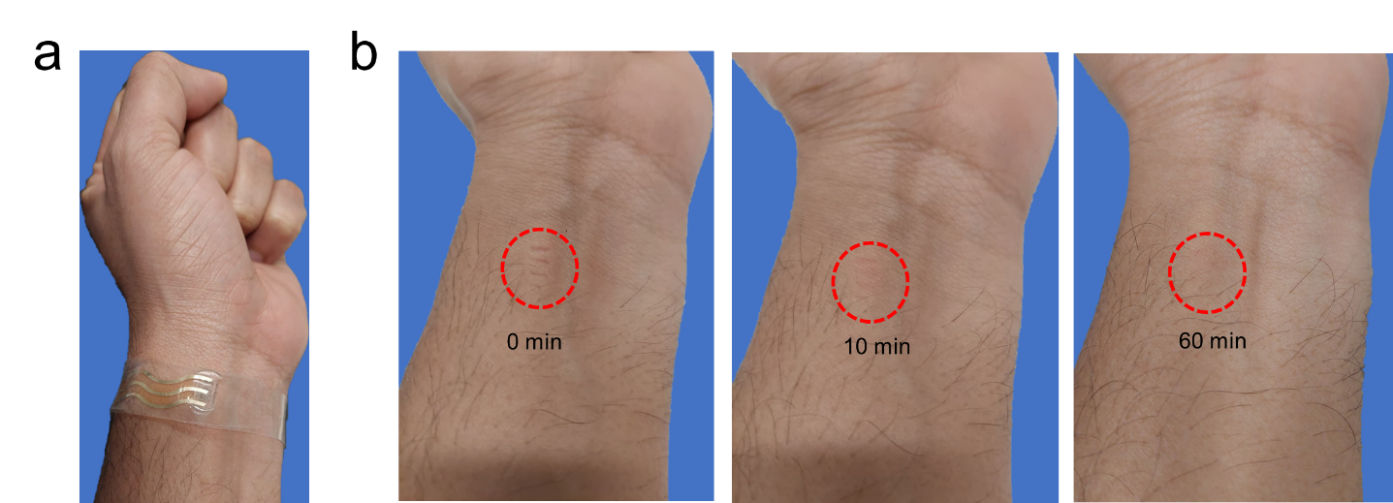


**Figure S12**. Skin reaction following sensor patch removal. (a) Photograph showing the sensor patch affixed to the wrist of a subject. (b) Time-course images demonstrating the visual impact on skin at the sensor application site (indicated by red circles) immediately after patch removal (0 min), 10 minutes post-removal, and 60 minutes post-removal. The initial erythema and indentation gradually resolve over the one-hour observation period.


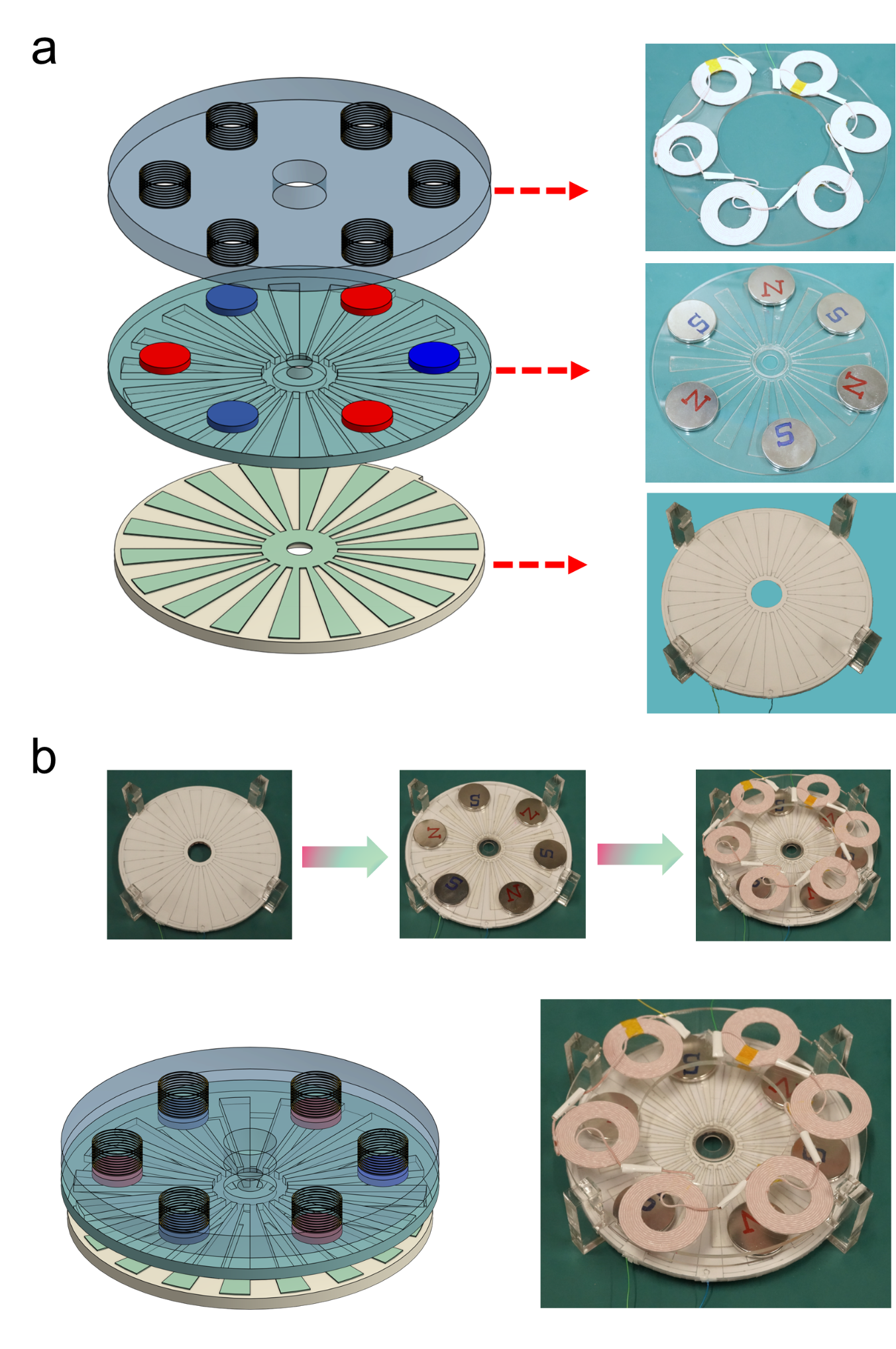


**Figure S13**. Fabrication and assembly of the hybrid power generation system (HPGS). (a) Layer-by-layer assembly sequence of TENG components with corresponding photographs. (b)The assembled device showing integration of TENG and EMG modules.


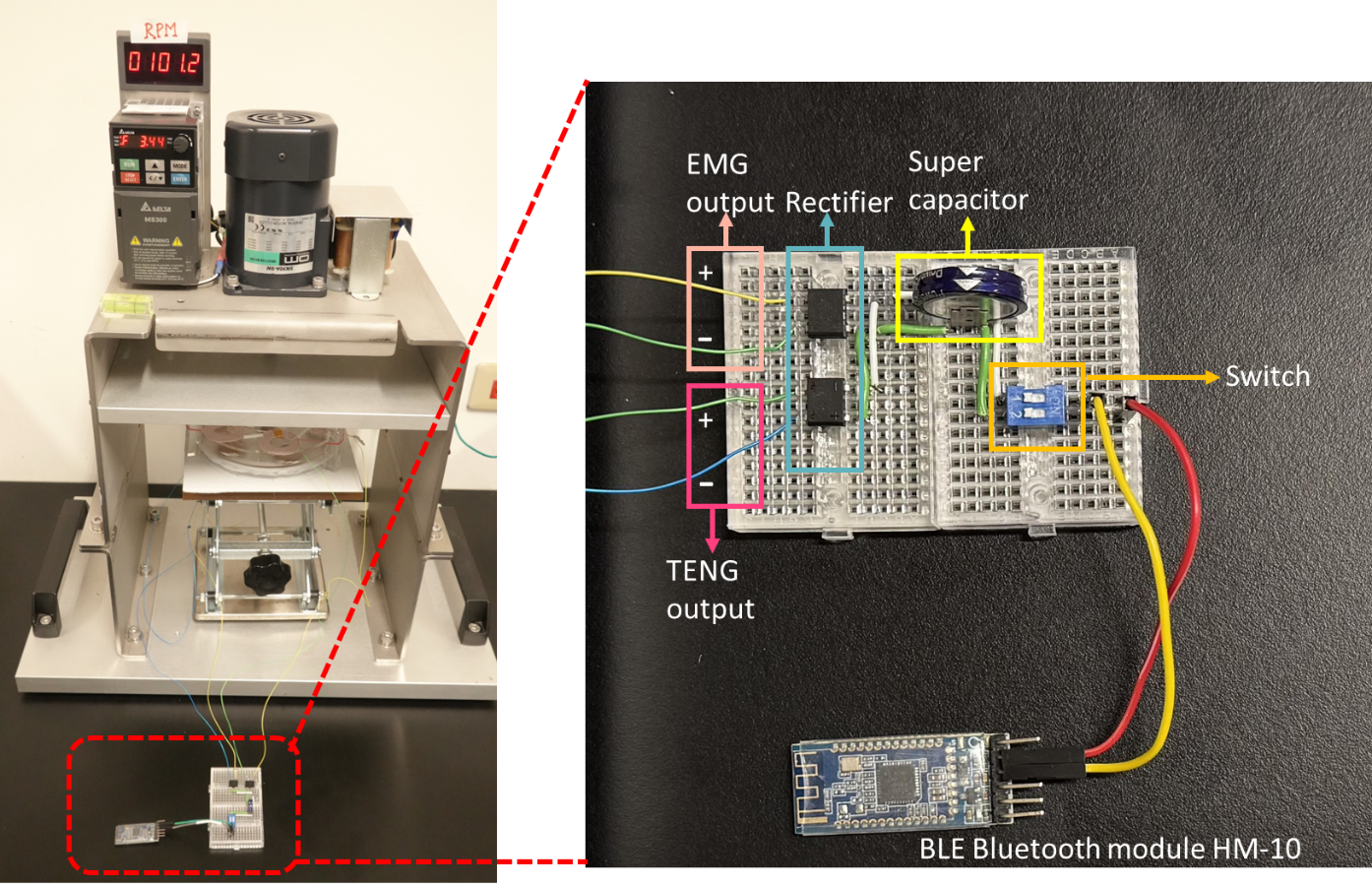


**Figure S14**. Experimental circuit configuration demonstrating supercapacitor charging and wireless BLE module functionality.


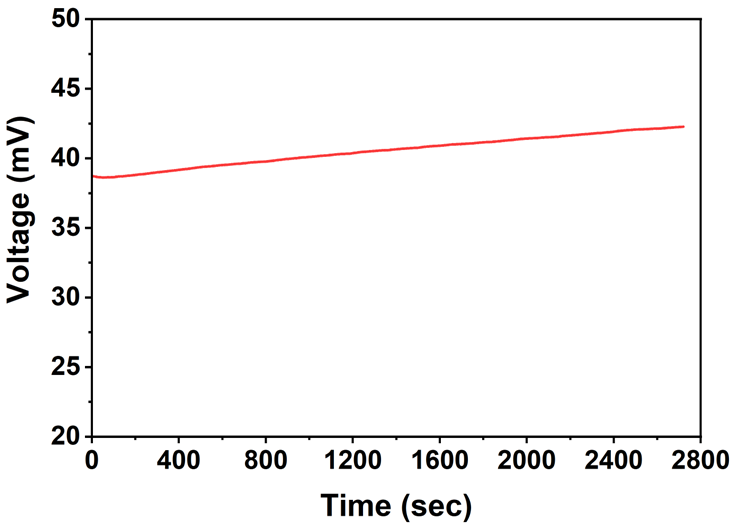


**Figure S15**. Potential variation of the as-fabricated reference electrode in simulated ISF, the open-circuit potential versus a commercial Ag/AgCl reference electrode (3M NaCl) was measured.


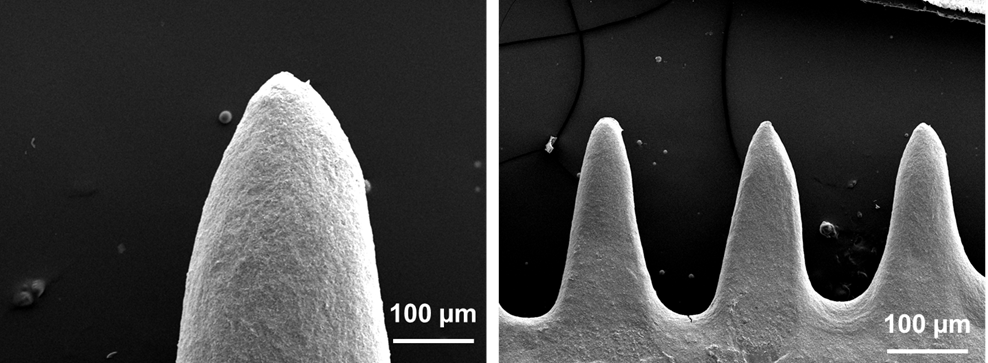


**Figure S16**. SEM images of the microneedles showing the full coverage of the needle tip.
